# Nonlocal-to-Local Convergence of Cahn–Hilliard Equations: Neumann Boundary Conditions and Viscosity Terms

**DOI:** 10.1007/s00205-020-01573-9

**Published:** 2020-10-04

**Authors:** Elisa Davoli, Luca Scarpa, Lara Trussardi

**Affiliations:** 1 Institut für Mathematik, University of Vienna, Oskar-Morgenstern-Platz 1, 1090 Vienna, Austria; 2 Institute of Analysis and Scientific Computing, TU Wien, Wiedner Hauptstrasse 8-10, 1040 Vienna, Austria

## Abstract

We consider a class of nonlocal viscous Cahn–Hilliard equations with Neumann boundary conditions for the chemical potential. The double-well potential is allowed to be singular (e.g. of logarithmic type), while the singularity of the convolution kernel does not fall in any available existence theory under Neumann boundary conditions. We prove well-posedness for the nonlocal equation in a suitable variational sense. Secondly, we show that the solutions to the nonlocal equation converge to the corresponding solutions to the local equation, as the convolution kernels approximate a Dirac delta. The asymptotic behaviour is analyzed by means of monotone analysis and Gamma convergence results, both when the limiting local Cahn–Hilliard equation is of viscous type and of pure type.

## Introduction

The aim of the present paper is to study the well-posedness and the asymptotic behaviour as $\varepsilon \searrow 0$ of a family of nonlocal viscous Cahn–Hilliard equations with Neumann boundary conditions in the following form:

1.1 $$\begin{aligned} \partial _t u_\varepsilon - \Delta \mu _\varepsilon =0 \qquad&\text {in } (0,T)\times \Omega \,,\end{aligned}$$

1.2 $$\begin{aligned} \mu _\varepsilon = \tau _\varepsilon \partial _t u_\varepsilon +(K_\varepsilon *1)u_\varepsilon -K_\varepsilon *u_\varepsilon +\Psi '(u_\varepsilon ) -g_\varepsilon \qquad&\text {in } (0,T)\times \Omega \,,\end{aligned}$$

1.3 $$\begin{aligned} \partial _\mathbf{n} \mu _\varepsilon =0\qquad&\text {on } (0,T)\times \partial \Omega \,,\end{aligned}$$

1.4 $$\begin{aligned} u_\varepsilon (0)=u_{0,\varepsilon } \qquad&\text {in } \Omega \,, \end{aligned}$$

where $\Omega$ is a smooth bounded domain in $\mathbb {R}^d$ ($d=2,3$), $T>0$ is a fixed final time, and $\Psi '$ represents the derivative a double-well potential. Moreover, $\varepsilon >0$ is a fixed parameter, $\tau _\varepsilon >0$ is a positive viscosity coefficient, $K_\varepsilon :\Omega \times \Omega \rightarrow \mathbb {R}$ is a suitable symmetric convolution kernel, and $g_\varepsilon$ represents a distributed forcing term. The variables $u_\varepsilon$ and $\mu _\varepsilon$ are referred to as “order parameter” and “chemical potential”, respectively.

The evolution problem (1.1)–(1.4) is related to the gradient flow (in the $H^{-1}$-metric) associated to a nonlocal free energy functional of the form

1.5 $$\begin{aligned} \mathcal E_{\varepsilon }(\varphi )&=\frac{1}{4} \int _{\Omega }\int _{\Omega }K_\varepsilon (x,y) |\varphi (x)-\varphi (y)|^{2}\,\text {d}x\,\text {d}y + \int _{\Omega }\Psi (\varphi (x))\,\text {d}x\,. \end{aligned}$$

Indeed, the contributions $(K_\varepsilon *1) u_\varepsilon - K_\varepsilon *u_\varepsilon + \Psi '(u_\varepsilon )$ in the definition of the chemical potential are obtained exactly from the (sub)differentiation of the functional (1.5). The extra term $\tau _\varepsilon \partial _t u_\varepsilon$ represents on the other side a viscosity regularization, acting on the dissipation of the system.

The analysis of nonlocal models dates back to the early 90’s, when Giacomin and Lebowitz investigated, in their seminal paper [36], a hydrodynamic limit of a microscopic model for a *d*-dimensional lattice gas evolving via a Poisson nearest-neighbor process. In that work, the authors derived a free energy functional in nonlocal form (1.5), and proposed the corresponding gradient flow to model phase-change in binary alloys. The viscous regularization in the definition of the chemical potential was originally introduced in the context of the local Cahn–Hilliard equation by Novick–Cohen in [50]. The mathematical literature on the nonlocal Cahn–Hilliard equation is widely developed: we can mention, among many others, the contributions [2, 5, 27, 28, 39] and the references therein.

The rapidly growing attention to the nonlocal Cahn–Hilliard equation is due on the one hand to its microscopic justification, and on the other hand to its connection with the corresponding local model. Indeed, at least in a formal way, the nonlocal dynamics approach the local ones when the family of interaction kernels $(K_\varepsilon )_\varepsilon$ concentrates around the origin. The main issue we assess in this paper is the asymptotic convergence of solutions to the nonlocal system (1.1)–(1.4) to the corresponding local one, as the data $(g_\varepsilon )_\varepsilon$ approximate a new source *g* and the coefficients $\tau _\varepsilon$ converge to a certain new viscosity parameter $\tau$. The local form of the limiting Cahn–Hilliard equation reads is

1.6 $$\begin{aligned} \partial _t u - \Delta \mu =0 \qquad&\text {in } (0,T)\times \Omega \,,\end{aligned}$$

1.7 $$\begin{aligned} \mu = \tau \partial _t u -\Delta u +\Psi '(u)-g \qquad&\text {in } (0,T)\times \Omega \,,\end{aligned}$$

1.8 $$\begin{aligned} \partial _\mathbf{n} u=0 \quad \text {and}\quad \partial _\mathbf{n} \mu =0\qquad&\text {on } (0,T)\times \partial \Omega \,,\end{aligned}$$

1.9 $$\begin{aligned} u(0)=u_{0} \qquad&\text {in } \Omega \,, \end{aligned}$$

where $\tau \geqq 0$ is the limiting viscosity parameter, which is allowed to vanish. The choices $\tau >0$ and $\tau =0$ correspond to the viscous case and pure case, respectively.

As its nonlocal counterpart, the local Cahn–Hilliard equation is related to the gradient flow in the $H^{-1}$ metric of the Ginzburg-Landau free energy functional

1.10 $$\begin{aligned} \mathcal E(\varphi )= \frac{1}{2}\int _{\Omega }|\nabla \varphi (x)|^2\,\text {d}x +\int _\Omega \Psi (\varphi (x)) \,\text {d}x\,, \end{aligned}$$

in the sense that the contribution $-\Delta u + \Psi '(u)$ results from the subdifferentiation of $\mathcal E$. Again, the viscosity term $\tau \partial _t u$ acts on the dissipation of the system: if $\tau =0$, one recovers the so-called *pure* Cahn–Hilliard equation, while if $\tau >0$ one obtains the *viscous* Cahn–Hilliard equation. In our analysis, the nonlocal viscosity coefficients $(\tau _\varepsilon )_\varepsilon$ are assumed to be strictly positive, while the local coefficient $\tau$ is allowed to vanish.

The local Cahn–Hilliard equation was first proposed in [9] in relation to phase-change in metallic alloys and to spinodal decomposition (see [44]). Nowadays, the model is a widely used in various contexts such as diffuse interface modelling in physics and biology, with several applications to tumor growth dynamics, image processing, and population dynamics. From the mathematical point of view, the local Cahn–Hilliard equation has been studied thoroughly in the last decades, also in much more complex settings. We mention, among many others, the works [11–13, 15, 16, 18, 37, 43] on well-posedness also under more general dynamic boundary conditions. Some studies on nonlinear viscosity contributions have been proposed in [6, 49, 56]. We also recall the contributions [14, 19, 20, 23, 40] dealing with optimal control problems, as well as [17, 22, 38] on asymptotics. The local Cahn–Hilliard equation has also been widely studied recently in connection to diffuse-interface models for fluid-dynamics: we refer to [1, 3, 10, 29, 30] and the references therein.

As has already been mentioned, the behaviour of the nonlocal Cahn–Hilliard equation “approaches” the one of the local equation when the family of convolution kernels is sufficiently peaked around 0. The study of nonlocal-to-local convergence of energy functionals in relation to Sobolev spaces theory had been carried out originally by by Bourgain et al. [7, 8], and by Mazy’a and Shaposhnikova [45, 46]. This asymptotic analysis was also extended by Ponce [51, 52], with studies on Gamma convergence and nonlocal Poincaré-type inequalities. A first criterion for the convergence of gradient flows from the Gamma-convergence of the respective energies was given by Sandier and Serfaty [55] in a abstract setting and for smooth energies, with applications to Ginzburg-Landau functionals (see also [42, 53, 57] for further details in this direction).

In particular, the above-mentioned results [51, 52] provide the pointwise convergence

$$\begin{aligned} \lim _{\varepsilon \searrow 0} \mathcal E_\varepsilon (\varphi )= \mathcal E(\varphi ) \qquad \forall \,\varphi \in H^1(\Omega ) \end{aligned}$$

as soon as the convolution kernels $(K_\varepsilon )_\varepsilon$ are chosen as

1.11 $$\begin{aligned} K_\varepsilon :\Omega \times \Omega \rightarrow [0,+\infty )\,, \qquad K_\varepsilon (x,y):=\frac{\rho _\varepsilon (|x-y|)}{|x-y|^2}\,, \quad x,y\in \Omega \,, \end{aligned}$$

where $(\rho _\varepsilon )_\varepsilon$ is a suitable family of mollifiers converging to a Dirac delta.

Building upon these variational convergences, in a previous contribution of ours [24] we rigorously derived some nonlocal-to-local asymptotics of solutions to Cahn–Hilliard equations in the setting of periodic boundary conditions and with no viscosity effects. The periodic setting adopted in [24] was fundamental to overcome the singular behaviour of the convolution kernel (1.11). Indeed, kernels in the form (1.11) do not possess any $W^{1,1}$ regularity (see for example [21, Remark 1]), which is the usual minimum requirement in the whole literature on nonlocal Cahn–Hilliard systems. This resulted in the impossibility of framing the nonlocal problem in any available existence theory, and required an ad-hoc analysis. In this direction, the arguments strongly relied on the assumption of periodic boundary conditions.

The results in [24] (see also [47] for a simpler case) are very satisfactory since they provide a novel contribution in the direction of local asymptotics of Cahn–Hilliard equations. Nevertheless, the most natural choice of boundary conditions in phase-field modelling if of no-flux type. Consequently, it is crucial in this direction to generalize the periodic framework to other settings more suited for applications. The nonlocal-to-local convergence of pure Cahn–Hilliard equations with Neumann boundary conditions was, to the authors’ knowledge, still an open problem. The main novelty of the present paper is to finally extend some rigorous nonlocal-to-local convergence results for Cahn–Hilliard equations to the case of homogeneous Neumann boundary conditions.

Let us briefly describe now the main difficulties arising in the case of Neumann boundary conditions. The first hurdle has been already anticipated and concerns the regularity of the convolution kernel. Indeed, in the form (1.11) the kernel $K_\varepsilon$ is not $W^{1,1}$, and not even $L^1$ in dimension $d=2$. This results in the necessity of rigorously formulate the nonlocal problem without relying on any available existence theory. The main idea here is that even if the convolution operator $\varphi \mapsto K_\varepsilon *\varphi$ may be ill-defined under (1.11), the nonlocal operator $B_\varepsilon :\varphi \mapsto (K_\varepsilon *1)\varphi -(K_\varepsilon *\varphi )$ appearing in the equation (1.2) can be rigorously defined instead.

The second main problem consists in the (im)possibility of proving space regularity for the solutions to the nonlocal equation (i.e. when $\varepsilon >0$ is fixed). If the convolution kernel is $W^{1,1}$ this follows directly from the properties of the convolution, i.e. formally shifting the gradient operator on the kernel as $\nabla (K_\varepsilon *u_\varepsilon ) =(\nabla K_\varepsilon ) * u_\varepsilon$. However, for singular kernels as in (1.11) this procedure fails. Under periodic boundary conditions (i.e. working on the *d*-dimensional flat torus) the main idea to overcome this problem was to use a certain integration-by-parts formula, which hinges in turn on some compatibility conditions between the convolution operator and the Laplace operator. More specifically, in [24] the periodic setting allowed to prove a (formal) relation in the form $\nabla (K_\varepsilon *u_\varepsilon )= K_\varepsilon *\nabla u_\varepsilon$, from which one could deduce $H^1$-regularity of the nonlocal solutions. Nevertheless, under Neumann boundary conditions (i.e. working on a bounded domain $\Omega \subset \mathbb {R}^d$), in order to prove an analogous compatibility relation one is forced to extend the nonlocal solution $u_\varepsilon$ to 0 outside $\Omega$. Clearly, $H^1$-regularity in $\Omega$ does not imply $H^1$-regularity on the whole $\mathbb {R}^d$ for such extension. This gives rise to several extra boundary contribution terms which blow up as the approximating parameter vanishes.

The main consequence is that in the case of Neumann boundary conditions one loses any $H^1$-estimate on the nonlocal solutions. It follows that the natural variational setting to frame the nonlocal problem (1.1)–(1.4) is not the usual one given by the triple $(H^1(\Omega ), L^2(\Omega ), H^1(\Omega )^*)$, but instead an abstract one $(V_\varepsilon , L^2(\Omega ), V_\varepsilon ^*)$, depending on $\varepsilon$, where $V_\varepsilon$ represents, roughly speaking, the domain of the nonlocal energy contribution in (1.5). As the inclusion $V_\varepsilon \hookrightarrow L^2(\Omega )$ is not compact, one loses any reasonable compactness property on the approximated solutions in order to pass to the limit in the nonlinearity. This issue is overcome by the introduction of the viscosity term $\tau _\varepsilon \partial _t u_\varepsilon$. Indeed, if $\tau _\varepsilon$ is strictly positive one can show “by hand” a strong convergence in $L^2(\Omega )$ for some regularized solutions, even without relying on any $H^1$ estimates.

The third main problem concerns the boundary conditions of Neumann type for *u* in the limiting local problem. Indeed, while the nonlocal system is of order 2 in space, hence it only needs one boundary condition (for the chemical potential), the limiting local equation is of order 4 in space and requires two boundary conditions instead: one for $\mu$ and one for *u*. One of the major point is to understand which is the natural extra boundary condition for *u*, and how this one emerges when $\varepsilon \searrow 0$. It is clear that the Neumann boundary condition for the chemical potential is preserved by the local asymptotics. On the other hand, the scenario for *u* is more subtle: the answer is implicitly given by studying the Gamma convergence of the nonlocal energies. Indeed, in [52] Ponce proved a Gamma convergence result in the form

$$\begin{aligned}&\lim _{\varepsilon \searrow 0}\frac{1}{4} \int _{\Omega }\int _{\Omega }K_\varepsilon (x,y) |\varphi _\varepsilon (x)-\varphi _\varepsilon (y)|^{2}\,\text {d}x\,\text {d}y\\&\qquad = {\left\{ \begin{array}{ll} \frac{1}{2}\int _\Omega |\nabla \varphi (x)|^2\,\text {d}x \quad &{}\text {if } \nabla \varphi \in L^2(\Omega )\,,\\ +\infty \quad &{}\text {otherwise}\,, \end{array}\right. } \end{aligned}$$

whenever $\varphi _\varepsilon \rightarrow \varphi$ in $L^2(\Omega )$. Note that the limiting energy contribution on the right-hand side is the potential associated to the negative Laplacian with homogeneous Neumann boundary conditions. Hence, this implicitly reveals that the “correct” choice of boundary condition arising for *u* in the local limit is of Neumann type. Such idea is indeed proved rigorously performing the local asymptotics on the variational formulation for the nonlocal problem (1.1)–(1.4). The advantage of working using a variational approach is that the boundary conditions are implicitly contained in the variational formulation itself, and they have not to be tracked explicitly performing a pointwise analysis on the boundary.

We are now in a position to present the two main theorems that we prove in this paper.

The first main result is the well-posedness for the nonlocal system (1.1)–(1.4) with Neumann boundary conditions when $\varepsilon >0$ is fixed. Here, the viscosity coefficient $\tau _\varepsilon$ is assumed to be strictly positive, the convolution kernel is of the form (1.11), and the double-well potential may be singular. In particular, we include in our analysis all the typical examples of polynomial, logarithmic, and double-obstacle potentials:

$$\begin{aligned}&\Psi _{pol}(r):=\frac{1}{4}(r^2-1)^2\,, \qquad r\in \mathbb {R}\,,\\&\Psi _{log}(r):= \frac{\vartheta }{2}\left[ (1+r)\ln (1+r) + (1-r)\ln (1-r) \right] - \frac{\vartheta _0}{2}\,, \\&\quad r\in (-1,1)\,, \quad 0<\vartheta <\vartheta _0\,,\\&\Psi _{doub}(r):= {\left\{ \begin{array}{ll} c(1-r^2) \quad &{}\text {if } r\in [-1,1]\,,\\ +\infty \quad &{}\text {otherwise}\,, \end{array}\right. } \qquad c>0\,. \end{aligned}$$

In view of this, the derivative of $\Psi$ is interpreted as a subdifferential in the sense of convex analysis, and equation (1.2) becomes a differential inclusion. The proof of well-posedness is based on a suitable approximation of the problem, given by a Yosida-type regularization on the nonlinearity and an additional elliptic local regularization in the chemical potential. A novel abstract variational setting $(V_\varepsilon , L^2(\Omega ), V_\varepsilon ^*)$ is introduced and uniform estimates on the approximated solutions are obtained. Using the viscous contribution in the chemical potential, strong compactness in $L^2$ is recovered even with no $H^1$-estimates on the solutions. Strong convergences are then proved and a passage to the limit provides solutions to the original nonlocal problem.

The second main result of this paper is the asymptotic analysis of the nonlocal system as $\varepsilon \searrow 0$. Here, we assume that the forcing terms $(g_\varepsilon )_\varepsilon$ converge to a certain source *g*, and that the viscosity coefficients satisfy

$$\begin{aligned} \lim _{\varepsilon \searrow 0}\tau _\varepsilon =\tau \,. \end{aligned}$$

Here, the coefficient $\tau$ is allowed to be nonnegative: when $\tau >0$ we obtain then nonlocal-to-local convergence of viscous Cahn–Hilliard equations, while if $\tau =0$ we obtain the local asymptotics of nonlocal viscous Cahn–Hilliard equations with vanishing viscosities. The proof is based on uniform estimates in $\varepsilon$ on the nonlocal solutions. Here, the strong compactness in $L^2$ is obtained by proving an ad-hoc compactness inequality involving the family on functional spaces $(V_\varepsilon )_{\varepsilon >0}$. The identification of the local limit $-\Delta u$ is obtained through the combination of monotone analysis techniques and Gamma-convergence results for the nonlocal energy functional (1.5).

We conclude by highlighting some possible applications of our results to phase-field modelling. The relevance of nonlocal-to-local convergence of Cahn–Hilliard equations with Neumann boundary conditions is significant: among many others, we can mention here possible connections with optimal control of tumor growth models. In the recent years, phase-field models have been widely used in tumor growth dynamics, both in the local case (see [25, 31–35] and the references therein) and in the nonlocal case (see [26] and [48, 54] for nonlocal Cahn–Hilliard equations with reaction terms). One of the main advantages of the nonlocal setting is that regularity results on the solutions are usually easier to obtain, not needing to rely on elliptic regularity properties. As a consequence, the availability of rigorous nonlocal-to-local convergence results would give the opportunity to approximate solutions to local phase-field systems with the solutions to the corresponding nonlocal ones, which are indeed simpler to handle on the mathematical side. For example, refined regularity on the solutions are fundamental when dealing with optimal control problems, in order to write first-order conditions for optimality. Hence, possible outcomes of nonlocal-to-local asymptotics concern refined analysis of optimal control of phase-field systems, in terms of passing to the (local) limit within first-order conditions for optimality for the nonlocal system.

The paper is structured in the following way: in Sect. 2 we state the assumptions, and we introduce the abstract variational settings. Section 3 is devoted to present the two main results. Section 4 contains the proof of well-posedness of the nonlocal system (1.1)–(1.4), while Sect. 5 focuses on the proof of nonlocal-to-local asymptotics.

## Mathematical Setting

### Assumptions

Throughout the paper, $\Omega$ is a smooth bounded domain in $\mathbb {R}^d$, with $d=2,3$, and $T>0$ is a fixed final time. We will use the notation $Q_t:=(0,t)\times \Omega$ for every $t\in (0,T]$, and set $Q:=Q_T$, and $\Sigma :=(0,T)\times \partial \Omega$. Moreover, $(\rho _\varepsilon )_{\varepsilon >0}$ is a family of mollifiers with the following properties (see [51, 52]):

$$\begin{aligned}&\rho _\varepsilon :\mathbb {R}\rightarrow [0,+\infty )\,, \qquad \rho _\varepsilon \in L^1_{loc}(\mathbb {R})\,, \qquad \rho _\varepsilon (r)=\rho _\varepsilon (-r) \quad \forall \,r\in \mathbb {R}\,, \qquad \forall \,\varepsilon>0\,;\\&\int _0^{+\infty } \rho _\varepsilon (r)r^{d-1}\,\text {d}r =\frac{2}{C_d} \quad \forall \,\varepsilon>0\,;\\&\lim _{\varepsilon \searrow 0}\int _\delta ^{+\infty } \rho _\varepsilon (r)r^{d-1}\,\text {d}r=0 \quad \forall \,\delta >0\,, \end{aligned}$$

where $C_d:=\int _{S^{d-1}} |e_1\cdot \sigma |^2\,\text {d}\mathcal H^{d-1}(\sigma )$. We define the family of convolution kernels as

2.1 $$\begin{aligned}&K_\varepsilon :\Omega \times \Omega \rightarrow [0,+\infty )\,, \qquad K_\varepsilon (x,y):=\frac{\rho _\varepsilon (|x-y|)}{|x-y|^2}\,, \nonumber \\&\quad \text {for a.e.}~x,y\in \Omega \,, \varepsilon >0\,. \end{aligned}$$

Throughout the paper, $\gamma :\mathbb {R}\rightarrow 2^\mathbb {R}$ is a maximal monotone graph with $0\in \gamma (0)$ and $\Pi :\mathbb {R}\rightarrow \mathbb {R}$ is $C_\Pi$-Lipschitz-continuous with $\Pi (0)=0$. It follows in particular that there exists a proper, convex, lower semicontinuous function $\hat{\gamma }:\mathbb {R}\rightarrow [0,+\infty ]$ with $\hat{\gamma }(0)=0$ and $\partial \hat{\gamma }=\gamma$ in the sense of convex analysis. Similarly, we set $\hat{\Pi }(s):=\int _0^s\Pi (r)\,\text {d}r$ for every $s\in \mathbb {R}$. With these notations, the double-well potential $\Psi$ entering the system is represented by the sum $\hat{\gamma }+ \hat{\Pi }$.

### Variational Setting and Preliminaries

We introduce the functional spaces

$$\begin{aligned} H:=L^2(\Omega )\,, \qquad V:=H^1(\Omega )\,, \qquad W:=\left\{ \varphi \in H^2(\Omega ): \partial _\mathbf{n}\varphi = 0 \text { a.e. on } \partial \Omega \right\} \,, \end{aligned}$$

endowed with their natural norms, and we identify *H* with its dual space in the usual way, so that

$$\begin{aligned} W \hookrightarrow V \hookrightarrow H \hookrightarrow V^* \hookrightarrow W^* \end{aligned}$$

where all the inclusions are continuous, dense, and compact. The Laplace operator with homogeneous Neumann conditions will be intended both as a bounded linear operator

$$\begin{aligned} -\Delta :V\rightarrow V^*\,, \qquad \langle -\Delta \varphi ,\zeta \rangle _V:= \int _\Omega \nabla \varphi (x) \cdot \nabla \zeta (x)\,\text {d}x\,, \quad \varphi ,\zeta \in V\,, \end{aligned}$$

and as unbounded linear operator on *H* with domain *W*. For every $\varphi \in V^*$, we use the notation $\varphi _\Omega :=\frac{1}{|\Omega |} \langle \varphi ,1\rangle _{V}$ for the mean value on $\Omega$. As a direct consequence of the Poincaré-Wirtinger inequality it holds that

$$\begin{aligned} -\Delta :\{\varphi \in V:\varphi _\Omega =0\}\rightarrow \{\varphi \in V^*:\varphi _\Omega =0\} \end{aligned}$$

is a linear isomorphism. We will denote its inverse by

$$\begin{aligned} \mathcal N:\{\varphi \in V^*:\varphi _\Omega =0\}\rightarrow \{\varphi \in V:\varphi _\Omega =0\}\,. \end{aligned}$$

For every $\varepsilon >0$, we set

$$\begin{aligned} V_\varepsilon&:= \left\{ \varphi \in L^2(\Omega ): \int _{\Omega }\int _\Omega K_\varepsilon (x,y)|\varphi (x)-\varphi (y)|^2\,\text {d}x\,\text {d}y <+\infty \right\} \,,\\ E_\varepsilon (\varphi )&:= \frac{1}{4}\int _{\Omega }\int _\Omega K_\varepsilon (x,y)|\varphi (x)-\varphi (y)|^2\,\text {d}x\,\text {d}y\,, \quad \varphi \in V_\varepsilon \,{.} \end{aligned}$$

Denoting by $a_\varepsilon :V_\varepsilon \times V_\varepsilon \rightarrow \mathbb {R}$ the natural bilinear form associated to $E_\varepsilon$,

$$\begin{aligned} a_\varepsilon (\varphi ,\psi ):= \frac{1}{2}\int _\Omega \int _\Omega K_\varepsilon (x,y) (\varphi (x)-\varphi (y)) (\psi (x)-\psi (y))\,\text {d}x\,\text {d}y, \quad \varphi ,\psi \in V_\varepsilon , \end{aligned}$$

we also define

$$\begin{aligned}&{W_\varepsilon }{:= \left\{ \varphi \in V_\varepsilon :\; \exists \, f\in L^2(\Omega ): \; a_\varepsilon (\varphi ,\psi )=\int _\Omega f(x)\psi (x)\,\text {d}x \text { for all }\psi \in V_\varepsilon \right\} }\,,\\&B_\varepsilon (\varphi )(x):= \int _{\Omega } K_\varepsilon (x,y)(\varphi (x)-\varphi (y))\,\text {d}y\,, \quad \text {for a.e. }x\in \Omega \,,\quad \varphi \in W_\varepsilon \,. \end{aligned}$$

Let us note that with such definitions the symmetry of $K_\varepsilon$ yields

$$\begin{aligned}&(B_\varepsilon (\varphi ), \psi )_H= \frac{1}{2}\int _\Omega \int _\Omega K_\varepsilon (x,y) (\varphi (x)\\&\qquad -\varphi (y)) (\psi (x)-\psi (y))\,\text {d}x\,\text {d}y\\&\quad = a_\varepsilon (\varphi ,\psi ) \quad \forall \,\varphi ,\psi \in W_\varepsilon \,. \end{aligned}$$

We point out that $E_\varepsilon :V_\varepsilon \rightarrow [0,+\infty )$ is convex and $B_\varepsilon : H\rightarrow H$ is a linear unbounded operator with domain $W_\varepsilon$. Additionally, we define the maps

$$\begin{aligned} \Vert \cdot \Vert _{V_\varepsilon }:V_\varepsilon \rightarrow [0,+\infty )\,, \qquad \Vert \cdot \Vert _{W_\varepsilon }:W_\varepsilon \rightarrow [0,+\infty ) \end{aligned}$$

as

$$\begin{aligned} \Vert \varphi \Vert _{V_\varepsilon }:=\sqrt{\Vert \varphi \Vert _H^2+ 2E_\varepsilon (\varphi )}\,, \qquad \Vert \varphi \Vert _{W_\varepsilon }:=\sqrt{\Vert \varphi \Vert _H^2 +\Vert B_\varepsilon (\varphi )\Vert _H^2}\,, \end{aligned}$$

and the bilinear forms

$$\begin{aligned} (\cdot ,\cdot )_{V_\varepsilon }:V_\varepsilon \times V_\varepsilon \rightarrow [0,+\infty )\,, \qquad (\cdot ,\cdot )_{W_\varepsilon }:W_\varepsilon \times W_\varepsilon \rightarrow [0,+\infty ) \end{aligned}$$

as

$$\begin{aligned} (\varphi _1,\varphi _2)_{V_\varepsilon }&:=(\varphi _1,\varphi _2)_H \\&\quad + \frac{1}{2}\int _\Omega \int _\Omega K_\varepsilon (x,y) (\varphi _1(x) -\varphi _1(y))(\varphi _2(x)-\varphi _2(y)) \,\text {d}x\,\text {d}y\,,\\ (\varphi _1,\varphi _2)_{W_\varepsilon }&:=(\varphi _1,\varphi _2)_H+ (B_\varepsilon (\varphi _1), B_\varepsilon (\varphi _2))_H\,. \end{aligned}$$

We collect some properties in the next lemma.

#### Lemma 1

The following properties hold for every $\varepsilon >0$:

1. The spaces $V_\varepsilon$ and $W_\varepsilon$, endowed with the norms $\Vert \cdot \Vert _{V_\varepsilon }$ and $\Vert \cdot \Vert _{W_\varepsilon }$, respectively, are complete.
2. The bilinear forms $(\cdot ,\cdot )_{V_\varepsilon }$ and $(\cdot ,\cdot )_{W_\varepsilon }$ are scalar products on $V_\varepsilon$ and $W_\varepsilon$ inducing the norms $\Vert \cdot \Vert _{V_\varepsilon }$ and $\Vert \cdot \Vert _{W_\varepsilon }$, respectively. In particular, $V_\varepsilon$ and $W_\varepsilon$ are Hilbert spaces.
3. For every $\sigma \in (0,1]$ we have $C^{0,\sigma }(\overline{\Omega })\hookrightarrow W_\varepsilon$ continuously, and there exists $C_{\varepsilon ,\sigma }>0$ such that
  $$\begin{aligned} B_\varepsilon (\varphi )\in L^\infty (\Omega )\,, \quad \Vert B_\varepsilon (\varphi )\Vert _{L^\infty (\Omega )}\leqq C_{\varepsilon ,\sigma }\Vert \varphi \Vert _{C^{0,\sigma }(\overline{\Omega })} \qquad \forall \,\varphi \in C^{0,\sigma }(\overline{\Omega })\,. \end{aligned}$$
4. The following inclusions are continuous and dense:
  $$\begin{aligned} W_\varepsilon \hookrightarrow V_\varepsilon \hookrightarrow H\,. \end{aligned}$$
  Moreover, $B_\varepsilon : \mathcal {D}(B_\varepsilon )\subset H\rightarrow H$, with $\mathcal {D}(B_\varepsilon )=W_\varepsilon$, is maximal monotone on *H*.
5. The unbounded linear operator $B_\varepsilon :H\rightarrow H$ extends to a bounded linear operator $B_\varepsilon :V_\varepsilon \rightarrow V_\varepsilon ^*$, and it holds that
  $$\begin{aligned} \Vert B_\varepsilon (\varphi )\Vert _{V_\varepsilon ^*}\leqq \Vert \varphi \Vert _{V_\varepsilon } \quad \forall \,\varphi \in V_\varepsilon \,. \end{aligned}$$
  Moreover, such extension coincides with the linear operator $A_\varepsilon :V_\varepsilon \rightarrow V_\varepsilon ^*$ associated to the bilinear form $a_\varepsilon$, defined as
  $$\begin{aligned} A_\varepsilon (\varphi ):=a_\varepsilon (\varphi ,\cdot )\,, \qquad \varphi \in V_\varepsilon \,. \end{aligned}$$
6. The map $E_\varepsilon :V_\varepsilon \rightarrow [0,+\infty )$ is of class $C^1$ and $DE_\varepsilon =B_\varepsilon :V_\varepsilon \rightarrow V_\varepsilon ^*$.

#### Proof

Step 1: properties *(1)–(2)*. It is clear that $\Vert \cdot \Vert _{V_\varepsilon }$ and $\Vert \cdot \Vert _{W_\varepsilon }$ are norms on $V_\varepsilon$ and $W_\varepsilon$, respectively. Let now $(y_n)_n$ be a Cauchy sequence in $V_\varepsilon$: then in particular it is a Cauchy sequence in *H*, so there exists $y\in H$ such that $y_n\rightarrow y$ in *H*. By lower semicontinuity it follows that $y\in V_\varepsilon$ as well, and that $y_n\rightarrow y$ in $V_\varepsilon$. A similar argument shows that $W_\varepsilon$ is complete as well. A direct computation shows that $(\cdot ,\cdot )_{V_\varepsilon }$ and $(\cdot ,\cdot )_{W_\varepsilon }$ are scalar products inducing the norms above.

Step 2: property *(3)*. For every $\varphi \in C^{0,\sigma }(\overline{\Omega })$, we have

$$\begin{aligned} |B_\varepsilon (\varphi (x))|\leqq \int _\Omega \rho _\varepsilon (|x-y|) \frac{|\varphi (x)-\varphi (y)|}{|x-y|^2}\,\text {d}y\leqq \Vert \varphi \Vert _{C^{0,\sigma }(\overline{\Omega })} \int _\Omega \frac{\rho _\varepsilon (|x-y|)}{|x-y|^{2-\sigma }}\text {d}y, \end{aligned}$$

where

$$\begin{aligned} \int _\Omega \frac{\rho _\varepsilon (|x-y|)}{|x-y|^{2-\sigma }}\text {d}y&= \int _{\Omega -x}\frac{\rho _\varepsilon (|z|)}{|z|^{2-\sigma }}\text {d}z \leqq \int _{\mathbb {R}^d} \frac{\rho _\varepsilon (|z|)}{|z|^{2-\sigma }}\text {d}z= \int _{\{|z|\leqq 1\}} \frac{\rho _\varepsilon (|z|)}{|z|^{2-\sigma }}\text {d}z\\&\quad +\int _{\{|z|>1\}} \frac{\rho _\varepsilon (|z|)}{|z|^{2-\sigma }}\text {d}z\\&\leqq \max _{|r|\leqq 1}\rho _\varepsilon (r)\int _{\{|z|\leqq 1\}} \frac{1}{|z|^{2-\sigma }}\text {d}z + \int _{\{|z|>1\}}\rho _\varepsilon (|z|)\,\text {d}z\,. \end{aligned}$$

The first term on the right-hand side is finite since $2-\sigma <d$, while the second term can be written as

$$\begin{aligned} |S^{d-1}|\int _1^{+\infty }\rho _\varepsilon (r)r^{d-1}\,\text {d}r<+\infty \end{aligned}$$

by the assumptions on $(\rho _\varepsilon )_\varepsilon$. The thesis follows by the arbitrariness of $x\in \Omega$.

Step 3: property *(4)*. First of all the fact that the inclusion $V_\varepsilon \hookrightarrow H$ is continuous is trivial by the definition of $\Vert \cdot \Vert _{V_\varepsilon }$. Second, for $\varphi \in W_\varepsilon$, a direct computation shows that

$$\begin{aligned} E_\varepsilon (\varphi )= & {} \frac{1}{4}\int _\Omega \int _\Omega K_\varepsilon (x,y) |\varphi (x)-\varphi (y)|^2\,\text {d}x\,\text {d}y\\= & {} \frac{1}{2}\int _\Omega B_\varepsilon (\varphi (x))\varphi (x)\,\text {d}x\\\leqq & {} \frac{1}{2}\Vert B_\varepsilon (\varphi )\Vert _H\Vert \varphi \Vert _H\,, \end{aligned}$$

so that $W_\varepsilon \hookrightarrow V_\varepsilon$ continuously. The density of $V_\varepsilon$ in *H* follows from the density of $C^{0,\sigma }(\overline{\Omega })$ in *H* and the fact that $C^{0,\sigma }(\overline{\Omega }) \subset W_\varepsilon \subset V_\varepsilon$.

The monotonicity of $B_\varepsilon$ is a direct consequence of its definition. We proceed by showing that it is maximal monotone. Let $\varphi \in H$. For every $\lambda ,\delta >0$ the elliptic problem

2.2 $$\begin{aligned} {\left\{ \begin{array}{ll} \varphi _{\delta \lambda } + \lambda \Delta ^2\varphi _{\delta \lambda } +\delta B_\varepsilon (\varphi _{\delta \lambda })=\varphi \quad &{}\text {in } \Omega \,,\\ \partial _\mathbf{n }\varphi _{\delta \lambda }= \partial _\mathbf{n }\Delta \varphi _{\delta \lambda }=0 \quad &{}\text {on } \partial \Omega \,, \end{array}\right. } \end{aligned}$$

admits a unique weak solution $\varphi _{\delta \lambda }\in W\hookrightarrow C^{0,1/4}(\overline{\Omega })\hookrightarrow W_\varepsilon$, in the sense that

$$\begin{aligned} (\varphi _{\delta \lambda },\psi )_H +\lambda (\Delta \varphi _{\delta \lambda }, \Delta \psi )_H +\delta (B_\varepsilon (\varphi _{\delta \lambda }), \psi )_H =(\varphi ,\psi )_H \qquad \forall \,\psi \in W\,. \end{aligned}$$

Taking arbitrary $\psi \in C^\infty _c(\Omega )$ in this variational formulation, we infer that $\Delta ^2\varphi _{\delta \lambda }\in H$ in the sense of distributions. Consequently, the classical elliptic regularity theory yields also that $\varphi _{\delta \lambda }\in H^4(\Omega )$, with $\partial _\mathbf{n }\Delta \varphi _{\delta \lambda }=0$ almost everywhere on $\partial \Omega$. Fix now $\delta >0$. Testing (2.2) by $\varphi _{\delta \lambda }$ and using the monotonicity of $B_\varepsilon$ and the Young inequality, it follows that

$$\begin{aligned} \Vert \varphi _{\delta \lambda }\Vert _H^2 +\lambda \Vert \Delta \varphi _{\delta \lambda }\Vert _H^2+ {2\delta E_\varepsilon (\varphi _{\delta \lambda })} \leqq \frac{1}{2}\Vert \varphi \Vert _H^2+ \frac{1}{2}\Vert \varphi _{\delta \lambda }\Vert _H^2 \quad \forall \,\lambda >0\,. \end{aligned}$$

Thus, there exists a positive constant *M* such that we have

$$\begin{aligned} {\Vert \varphi _{\delta \lambda }\Vert _{V_\varepsilon }^2} +\lambda \Vert \Delta \varphi _{\delta \lambda }\Vert _H^2 \leqq M \quad \forall \,\lambda >0\,. \end{aligned}$$

Noting that for all $\zeta \in V_\varepsilon$, by the symmetry of the kernel $K_\varepsilon$ and the Hölder inequality we have

$$\begin{aligned} \left( B_\varepsilon (\varphi _{\delta \lambda }),\zeta \right) _H= & {} \frac{1}{2}\int _\Omega \int _\Omega K_\varepsilon (x,y)(\varphi _{\delta \lambda }(x) -\varphi _{\delta \lambda }(y))(\zeta (x) -\zeta (y)) \,\text {d}x\,\text {d}y\\\leqq & {} 2\Vert \varphi _{\delta \lambda }\Vert _{V_\varepsilon } \Vert \zeta \Vert _{V_\varepsilon }\,, \end{aligned}$$

the estimates just proved ensure also that

$$\begin{aligned} \Vert B_\varepsilon (\varphi _{\delta \lambda })\Vert _{V_\varepsilon ^*}\leqq M\,. \end{aligned}$$

We infer that there exist $\varphi _\delta \in V_\varepsilon$ and $\eta _\delta \in V_\varepsilon ^*$ such that, as $\lambda \searrow 0$, $\lambda \varphi _{\delta \lambda }\rightarrow 0$ in *W*, $\varphi _{\delta \lambda }\rightharpoonup \varphi _\delta$ in $V_\varepsilon$, and $B_\varepsilon (\varphi _{\delta \lambda }) \rightharpoonup \eta _\delta$ in $V_\varepsilon ^*$. It follows that

$$\begin{aligned} (\varphi _\delta , \zeta )_H +\delta \langle \eta _\delta ,\zeta \rangle _{V_\varepsilon ^*,V_\varepsilon } =(\varphi , \zeta )_H \qquad \forall \,\zeta \in V_\varepsilon \,. \end{aligned}$$

Now, for all $\zeta \in V_\varepsilon$, by the symmetry of $B_\varepsilon$ and the bilinearity of $a_\varepsilon$ if holds that

$$\begin{aligned} \langle \eta _\delta ,\zeta \rangle _{V_\varepsilon ^*,V_\varepsilon } =\lim _{\lambda \rightarrow 0} (B_\varepsilon (\varphi _{\delta \lambda }),\zeta )_H= \lim _{\lambda \rightarrow 0} a_\varepsilon (\varphi _{\delta \lambda },\zeta )= a_\varepsilon (\varphi _\delta , \zeta )\,. \end{aligned}$$

This shows that

$$\begin{aligned} \delta a_\varepsilon (\varphi _\delta , \zeta )= (\varphi - \varphi _\delta , \zeta )_H \qquad \forall \,\zeta \in V_\varepsilon \,, \end{aligned}$$

so we conclude that $\varphi _\delta \in W_\varepsilon$ and $\eta _\delta =B_\varepsilon (\varphi _\delta )$. Hence,

2.3 $$\begin{aligned} \varphi _\delta + \delta B_\varepsilon (\varphi _\delta ) = \varphi \qquad \forall \,\delta >0\,. \end{aligned}$$

This proves that $B_\varepsilon$ is a maximal monotone operator on *H* (see [4, Thm. 2.2]). Testing now (2.3) by $\varphi _\delta$ and using Young inequality it is immediate to see that

2.4 $$\begin{aligned} \frac{1}{2}\Vert \varphi _\delta \Vert _H^2 + \delta {a_\varepsilon (\varphi _\delta , \varphi _\delta )}\leqq \frac{1}{2}\Vert \varphi \Vert _H^2\,. \end{aligned}$$

Let us note that since we have just proved that $\varphi _\delta \in W_\varepsilon$, in particular we have that $B_\varepsilon (\varphi _\delta )\in H$. Hence, if additionally $\varphi \in V_\varepsilon$, testing (2.3) by $B_\varepsilon (\varphi _\delta )$ and using Hölder and Young inequalities yields

2.5 $$\begin{aligned} 2 E_\varepsilon (\varphi _\delta ) + \delta \Vert B_\varepsilon (\varphi _\delta )\Vert _H^2&= {a_\varepsilon (\varphi _\delta , \varphi _\delta )} + \delta \Vert B_\varepsilon (\varphi _\delta )\Vert _H^2 =(B_\varepsilon (\varphi _\delta ), \varphi )_H\nonumber \\&=\frac{1}{2}\int _\Omega \int _\Omega K_\varepsilon (x,y)(\varphi _\delta (x)-\varphi _\delta (y)) (\varphi (x)-\varphi (y))\,\text {d}x\,\text {d}y\nonumber \\&\leqq 2 \sqrt{E_\varepsilon (\varphi )}\sqrt{E_\varepsilon (\varphi _\delta )}\leqq E_\varepsilon (\varphi _\delta ) + E_\varepsilon (\varphi )\,. \end{aligned}$$

We deduce that, as $\delta \searrow 0$, $\delta B_\varepsilon (\varphi _\delta )\rightarrow 0$ in *H*. Hence, by (2.3), $\varphi _\delta \rightarrow \varphi$ in *H*. By combining (2.4) and (2.5), we obtain that $\Vert \varphi _\delta \Vert _{V_\varepsilon }\leqq \Vert \varphi \Vert _{V_\varepsilon }$ for every $\delta >0$. As $V_\varepsilon$ is uniformly convex, this implies that $\varphi _\delta \rightarrow \varphi$ in $V_\varepsilon$, so that $W_\varepsilon \hookrightarrow V_\varepsilon$ densely.

Step 4: property *(5)*. For every $\varphi \in W_\varepsilon$ and $\zeta \in V_\varepsilon$, by the Hölder inequality we have

$$\begin{aligned} \left( B_\varepsilon (\varphi ),\zeta \right) _H= & {} \frac{1}{2}\int _\Omega \int _\Omega K_\varepsilon (x,y)(\varphi (x)-\varphi (y))(\zeta (x) -\zeta (y)) \,\text {d}x\,\text {d}y\\\leqq & {} 2\sqrt{E_\varepsilon (\varphi )}\sqrt{E_\varepsilon (\zeta )}\,. \end{aligned}$$

This implies that for every $\varphi \in W_\varepsilon$, the operator

$$\begin{aligned} \zeta \mapsto (B_\varepsilon (\varphi ),\zeta )_H\,, \quad \zeta \in V_\varepsilon \,, \end{aligned}$$

is linear and continuous on $V_\varepsilon$, and such that

$$\begin{aligned} \Vert \zeta \mapsto (B_\varepsilon (\varphi ),\zeta )_H\Vert _{V_\varepsilon ^*}\leqq \Vert \varphi \Vert _{V_\varepsilon } \qquad \forall \,\varphi \in W_\varepsilon \,. \end{aligned}$$

Since $W_\varepsilon \hookrightarrow V_\varepsilon$ is dense, we deduce that $B_\varepsilon$ extends to a bounded linear operator from $V_\varepsilon$ to $V_\varepsilon ^*$, and the thesis follows.

Step 5: property *(6)*. We observe that $E_\varepsilon :V_\varepsilon \rightarrow [0,+\infty )$ is convex and lower semicontinuous. A direct computation also shows that $DE_\varepsilon =B_\varepsilon$ in the sense of Gâteaux: since $B_\varepsilon :V_\varepsilon \rightarrow V_\varepsilon ^*$ is linear and continuous, the thesis follows. $\quad \square$

The next lemma shows some boundedness properties of the family $(B_\varepsilon )_\varepsilon$, uniformly in $\varepsilon$.

#### Lemma 2

The following inclusion is continuous:

$$\begin{aligned} V\hookrightarrow V_\varepsilon \,, \end{aligned}$$

and there exists a constant *C*, independent of $\varepsilon$, such that

$$\begin{aligned} \Vert \varphi \Vert _{V_\varepsilon }\leqq C\Vert \varphi \Vert _{V} \quad \forall \,\varphi \in V\,. \end{aligned}$$

For every $\varphi ,\zeta \in V$, if holds that

2.6 $$\begin{aligned} \lim _{\varepsilon \searrow 0}E_\varepsilon (\varphi )= \frac{1}{2}\int _\Omega |\nabla \varphi (x)|^2\text {d}x\,,\qquad \lim _{\varepsilon \searrow 0}\langle B_\varepsilon (\varphi _1), \varphi _2\rangle _{V_\varepsilon }= \int _\Omega \nabla \varphi _1(x)\cdot \nabla \varphi _2(x)\,\text {d}x\,. \end{aligned}$$

Finally, for every $\varphi \in H$ and for every sequence $(\varphi _\varepsilon )_{\varepsilon >0}\subset H$ with $\varphi _\varepsilon \rightarrow \varphi$ in *H*, we have

$$\begin{aligned} \liminf _{\varepsilon \searrow 0}E_\varepsilon (\varphi _\varepsilon ) \geqq E(\varphi ):= {\left\{ \begin{array}{ll} \frac{1}{2}\int _\Omega |\nabla \varphi (x)|^2\,\text {d}x \quad &{}\text {if } \varphi \in V\,,\\ +\infty &{}\text {if } \varphi \in H\setminus V\,. \end{array}\right. }\, \end{aligned}$$

In other words, $(E_\varepsilon )_{\varepsilon >0}$ $\Gamma$-converges to *E* with respect to the norm-topology of *H*.

#### Proof

By [52], there is a constant $C>0$ independent of $\varepsilon$ such that

$$\begin{aligned} E_\varepsilon (\varphi )\leqq C\Vert \nabla \varphi \Vert _H^2 \qquad \forall \,\varphi \in V\,, \end{aligned}$$

from which the first part of the thesis follows directly. The first limit in (2.6) is also a direct consequence of [52], the second limit in (2.6) can be proved by choosing $\varphi =\varphi _1\pm \varphi _2$ in the first limit.

Finally, by the $\Gamma$-convergence result in [51, Thm. 8], we know that

$$\begin{aligned} \liminf _{\varepsilon \searrow 0}E_\varepsilon (\varphi _\varepsilon )\geqq {\text {sc}}{-} \tilde{E}(\varphi )\,, \end{aligned}$$

where ${\text {sc}}{-} \tilde{E}$ is the lower semicontinuous envelope of

$$\begin{aligned} \tilde{E}:H\rightarrow [0,+\infty ]\,, \qquad \tilde{E}(\varphi ):= {\left\{ \begin{array}{ll} \frac{1}{2}\int _\Omega |\nabla \varphi (x)|^2\,\text {d}x \quad &{}\text {if } \varphi \in C^1(\overline{\Omega })\,,\\ +\infty &{}\text {otherwise }\,, \end{array}\right. } \end{aligned}$$

i.e.

$$\begin{aligned} {\text {sc}}{-} \tilde{E}(\varphi )= \inf \left\{ \liminf _{n\rightarrow \infty } \tilde{E}(\zeta _n): \zeta _n\rightarrow \varphi \quad \text {in } H\right\} \,. \end{aligned}$$

It is a standard matter to check that ${\text {sc}}{-} \tilde{E}=E$, so that the thesis follows. $\quad \square$

The last result of this section is a compactness criterion involving the family of operators $(E_\varepsilon )_\varepsilon$. The following lemma is fundamental as we do not have any compactness properties for the inclusions of the spaces $V_\varepsilon$ and $W_\varepsilon$. For the proof we refer to [24, Lemma. 4].

#### Lemma 3

For every $\delta >0$ there exist two constants $C_{\delta }>0$ and $\varepsilon _\delta >0$ such that, for every sequence $(\varphi _\varepsilon )_{\varepsilon \in (0,\varepsilon _\delta )}\subset V_\varepsilon$ if holds that

$$\begin{aligned} \Vert \varphi _{\varepsilon _1}-\varphi _{\varepsilon _2}\Vert ^2_{H}\leqq \delta \left( E_{\varepsilon _1}(\varphi _{\varepsilon _1}) + E_{\varepsilon _2}(\varphi _{\varepsilon _2})\right) +C_{\delta } \Vert \varphi _{\varepsilon _1}-\varphi _{\varepsilon _2}\Vert ^2_{V^*} \quad \forall \,\varepsilon _1,\varepsilon _2\in (0,\varepsilon _\delta )\,. \end{aligned}$$

## Main Results

Before stating our main results, we recall that the local Cahn–Hilliard equation is well-posed in the following sense:

### Theorem 3.1

Let $$\tau \geqq 0$$ and

3.1 $$\begin{aligned}&u_0 \in V\,, \qquad \hat{\gamma }(u_0)\in L^1(\Omega )\,, \qquad (u_0)_\Omega \in {\text {Int}}D(\gamma )\,,\end{aligned}$$

3.2 $$\begin{aligned}&g\in L^2(0,T; H)\,, \qquad g\in H^1(0,T; H) \quad \text {if } \tau =0\,. \end{aligned}$$

Then, there exists a triple $(u,\mu ,\xi )$ such that

3.3 $$\begin{aligned}&u \in H^1(0,T; V^*) \cap L^\infty (0,T; V) \cap L^2(0,T; W)\,,\qquad \tau u \in H^1(0,T; H)\,,\end{aligned}$$

3.4 $$\begin{aligned}&\mu \in L^2(0,T; V)\,, \qquad \tau \mu \in L^2(0,T; W)\,,\end{aligned}$$

3.5 $$\begin{aligned}&\xi \in L^2(0,T; H)\,, \qquad \xi \in \gamma (u) \quad \text {a.e. in } Q\,,\end{aligned}$$

3.6 $$\begin{aligned}&\partial _t u - \Delta \mu = 0 \quad \text {in } V^*\,, \quad \text {a.e. in } (0,T)\,,\end{aligned}$$

3.7 $$\begin{aligned}&\mu =\tau \partial _t u - \Delta u + \xi + \Pi (u) - g \quad \text {a.e. in } Q\,,\end{aligned}$$

3.8 $$\begin{aligned}&u(0)=u_0 \quad \text {a.e. in } \Omega \,. \end{aligned}$$

Moreover, the solution component *u* is unique, and the solution components $\mu$ and $\xi$ are unique if $\gamma$ is single-valued.

### Proof

We refer to [18] for a proof in a more general setting. $\quad \square$

The first result of this paper is the well-posedness of the nonlocal viscous Cahn–Hilliard equation complemented by Neumann boundary conditions for the chemical potential.

### Theorem 3.2

Let $\varepsilon >0$ and $\tau _\varepsilon >0$ be fixed. Then for every $(u_{0,\varepsilon },g_\varepsilon )$ with

3.9 $$\begin{aligned}&u_{0,\varepsilon } \in V_\varepsilon \,, \qquad \hat{\gamma }(u_{0,\varepsilon })\in L^1(\Omega )\,, \qquad (u_{0,\varepsilon })_\Omega \in {\text {Int}}D(\gamma )\,,\end{aligned}$$

3.10 $$\begin{aligned}&g_\varepsilon \in L^2(0,T; H)\,, \end{aligned}$$

there exists a triple $(u_\varepsilon ,\mu _\varepsilon ,\xi _\varepsilon )$ such that

3.11 $$\begin{aligned}&u_\varepsilon \in H^1(0,T; H) \cap L^\infty (0,T; V_\varepsilon ) \cap L^2(0,T; W_\varepsilon )\,,\end{aligned}$$

3.12 $$\begin{aligned}&\mu _\varepsilon \in L^2(0,T; W)\,,\end{aligned}$$

3.13 $$\begin{aligned}&\xi _\varepsilon \in L^2(0,T; H)\,, \qquad \xi _\varepsilon \in \gamma (u_\varepsilon ) \quad \text {a.e. in } Q\,,\end{aligned}$$

3.14 $$\begin{aligned}&\partial _t u_\varepsilon - \Delta \mu _\varepsilon = 0 \quad \text {a.e. in } Q\,,\end{aligned}$$

3.15 $$\begin{aligned}&\mu _\varepsilon =\tau _\varepsilon \partial _t u_\varepsilon + B_\varepsilon (u_\varepsilon ) +\xi _\varepsilon +\Pi (u_\varepsilon ) -g_\varepsilon \quad \text {a.e. in } Q\,,\end{aligned}$$

3.16 $$\begin{aligned}&u_\varepsilon (0)=u_{0,\varepsilon } \quad \text {a.e. in } \Omega \,. \end{aligned}$$

Furthermore, there exists a positive constant $M_\varepsilon$ such that, for every sets of data $(u_{0,\varepsilon }^1, g_\varepsilon ^1)$ and $(u_{0,\varepsilon }^2,g_\varepsilon ^2)$ satisfying (3.9)–(3.10), with $(u_{0,\varepsilon }^1)_\Omega =(u_{0,\varepsilon }^2)_\Omega$, and for every respective solutions $(u_\varepsilon ^1, \mu _\varepsilon ^1, \xi _\varepsilon ^1)$ and $(u_\varepsilon ^2, \mu _\varepsilon ^2, \xi _\varepsilon ^2)$ satisfying (3.11)–(3.16), it holds

$$\begin{aligned}&\Vert u_\varepsilon ^1-u_\varepsilon ^2\Vert _{C^0([0,T]; V^*)}^2+\tau _\varepsilon \Vert u_\varepsilon ^1-u_\varepsilon ^2\Vert _{C^0([0,T]; H)}^2 +\Vert E_\varepsilon (u_\varepsilon ^1-u_\varepsilon ^2)\Vert _{L^1(0,T)}\\&\qquad \leqq M_\varepsilon \left( \Vert u_{0,\varepsilon }^1-u_{0,\varepsilon }^2\Vert _{V^*}^2+\tau _\varepsilon \Vert u_{0,\varepsilon }^1-u_{0,\varepsilon }^2\Vert _{H}^2 +\Vert g_\varepsilon ^1-g_\varepsilon ^2\Vert _{L^2(0,T; V^*)}^2\right) \,. \end{aligned}$$

In particular, the solution component $u_\varepsilon$ is unique, and the solution components $\mu _\varepsilon$ and $\xi _\varepsilon$ are unique if $\gamma$ is single-valued.

Our second contribution concerns the nonlocal-to-local convergence. In particular, we show that, under suitable assumptions on the initial data $(u_{0,\varepsilon })_{\varepsilon }$ and on the forcing terms $(g_\varepsilon )_\varepsilon$, if the viscosities $(\tau _\varepsilon )_\varepsilon$ converge to a coefficient $\tau \geqq 0$, then the solutions to the respective viscous nonlocal Cahn–Hilliard equations converge, in suitable topologies, to the solutions to the limiting local Cahn–Hilliard equation with viscosity parameter $\tau \geqq 0$. Note that the viscosities $(\tau _\varepsilon )_\varepsilon$ are required to be strictly positive for all $\varepsilon >0$, whereas the limiting viscosity parameter $\tau$ is also allowed to vanish. Hence, such result has a duplex formulation. Indeed, if $\tau >0$ this shows the asymptotic convergence of the nonlocal *viscous* equation to the corresponding local *viscous* equation, while if $\tau =0$ this proves the approximability of solutions to the local *pure* equation by solutions to nonlocal equations with vanishing viscosities.

### Theorem 3.3

Assume that

$$\begin{aligned} \tau \geqq 0\,,\qquad (\tau _\varepsilon )_{\varepsilon >0}\subset (0,+\infty )\,, \qquad \lim _{\varepsilon \searrow 0}\tau _\varepsilon =\tau \,. \end{aligned}$$

Let the data $(u_0,g)$ satisfy (3.1)–(3.2), and let the family $(u_{0,\varepsilon }, g_\varepsilon )_{\varepsilon >0}$ satisfy (3.9)–(3.10) for all $\varepsilon >0$. Assume also that there exists $\varepsilon _0>0$ such that

3.17 $$\begin{aligned}&\sup _{\varepsilon \in (0,\varepsilon _0)}\left( \Vert u_{0,\varepsilon }\Vert _{V_\varepsilon }^2 + \Vert \hat{\gamma }(u_{0,\varepsilon })\Vert _{L^1(\Omega )} \right) <+\infty \,,\end{aligned}$$

3.18 $$\begin{aligned}&(g_\varepsilon )_{\varepsilon \in (0,\varepsilon _0)}\subset H^1(0,T; H) \quad \text {and}\quad \sup _{\varepsilon \in (0,\varepsilon _0)} \Vert g_\varepsilon \Vert _{H^1(0,T; H)}^2<+\infty \quad \text {if } \tau =0\,,\end{aligned}$$

3.19 $$\begin{aligned}&\exists \,[a_0,b_0]\subset {\text {Int}}D(\gamma ):\quad a_0\leqq (u_{0,\varepsilon })_\Omega \leqq b_0 \quad \forall \,\varepsilon \in (0,\varepsilon _0)\,,\end{aligned}$$

3.20 $$\begin{aligned}&u_{0,\varepsilon }\rightharpoonup u_0 \quad \text {in } H \quad \text {as } \varepsilon \searrow 0\,,\qquad g_\varepsilon \rightharpoonup g \quad \text {in } L^2(0,T; H) \quad \text {as } \varepsilon \searrow 0\,. \end{aligned}$$

Let $(u_\varepsilon ,\mu _\varepsilon ,\xi _\varepsilon )_{\varepsilon \in (0,\varepsilon _0)}$ be a family of solutions to (3.11)–(3.16) corresponding to the data $(u_{0,\varepsilon }, g_\varepsilon )$ and viscosity $\tau _\varepsilon$, where $u_\varepsilon$ is uniquely determined. Then, there exists a solution $(u,\mu ,\xi )$ to (3.3)–(3.8) corresponding to the data $(u_0,g)$ and viscosity $\tau$, where *u* is uniquely determined, such that, as $\varepsilon \searrow 0$,

$$\begin{aligned} u_\varepsilon \rightarrow u \qquad&\text {in } C^0([0,T]; H)\,,\\ \partial _t u_\varepsilon \rightharpoonup \partial _t u \qquad&\text {in } L^2(0,T;V^*)\,,\\ \partial _t u_\varepsilon \rightharpoonup \partial _t u \qquad&\text {in } L^2(0,T;H) \quad \text {if } \tau>0\,,\\ \tau _\varepsilon \partial _t u_\varepsilon \rightarrow 0 \qquad&\text {in } L^2(0,T;H) \quad \text {if } \tau =0\,,\\ \mu _\varepsilon \rightharpoonup \mu \qquad&\text {in } L^2(0,T; V)\,,\\ \mu _\varepsilon \rightharpoonup \mu \qquad&\text {in } L^2(0,T; W) \quad \text {if } \tau >0\,,\\ \xi _\varepsilon \rightharpoonup \xi \qquad&\text {in } L^2(0,T; H)\,. \end{aligned}$$

## Proof of Theorem 3.2

This section is devoted to the proof of well-posedness of the nonlocal viscous Cahn–Hilliard equation. Throughout the section, $\varepsilon >0$ and $\tau _\varepsilon >0$ are fixed.

### Approximation

For every $\lambda >0$, let $\gamma _\lambda :\mathbb {R}\rightarrow \mathbb {R}$ be the Yosida approximation of $\gamma$, having Lipschitz constant $1/\lambda$, and set $\hat{\gamma }_\lambda (s):=\int _0^s\gamma _\lambda (r)\,\text {d}r$ for every $s\in \mathbb {R}$. We consider the approximated problem

4.1 $$\begin{aligned} \partial _t u_\varepsilon ^\lambda - \Delta \mu _\varepsilon ^\lambda = 0 \qquad&\text {in } Q\,,\end{aligned}$$

4.2 $$\begin{aligned} \mu _\varepsilon ^\lambda = \tau _\varepsilon \partial _t u_\varepsilon ^\lambda -\lambda \Delta u_\varepsilon ^\lambda +B_\varepsilon (u_\varepsilon ^\lambda ) +\gamma _\lambda (u_\varepsilon ^\lambda ) +\Pi (u_\varepsilon ^\lambda )-g_\varepsilon \qquad&\text {in } Q\,,\end{aligned}$$

4.3 $$\begin{aligned} \partial _\mathbf{n}u_\varepsilon ^\lambda = \partial _\mathbf{n}\mu _\varepsilon ^\lambda = 0 \qquad&\text {in } \Sigma \,,\end{aligned}$$

4.4 $$\begin{aligned} u_\varepsilon ^\lambda (0)=u_{0,\varepsilon }^\lambda \qquad&\text {in } \Omega \,, \end{aligned}$$

where the initial datum $u_{0,\varepsilon }^\lambda$ satisfies

4.5 $$\begin{aligned}&u_{0,\varepsilon }^\lambda \in V\,, \qquad u_{0,\varepsilon }^\lambda \rightarrow u_{0,\varepsilon } \quad \text {in } H \quad \text {as } \varepsilon \searrow 0\,,\end{aligned}$$

4.6 $$\begin{aligned}&\sup _{\lambda \in (0,\lambda _0)}\left( \lambda \Vert u_{0,\varepsilon }^\lambda \Vert _{V}^2+ \Vert \hat{\gamma }(u_{0,\varepsilon }^\lambda )\Vert _{L^1(\Omega )} \right) <+\infty \end{aligned}$$

for a certain $\lambda _0>0$ (possibly depending on $\varepsilon$). The existence of an approximating sequence $(u_{0,\varepsilon }^\lambda )_\lambda$ satisfying (4.5)–(4.6) is guaranteed by assumption (3.1): for example, one can check that the classical elliptic regularization given by the unique solution to the problem

$$\begin{aligned} {\left\{ \begin{array}{ll} u_{0,\varepsilon }^\lambda - \lambda \Delta u_{0,\varepsilon }^\lambda = u_{0,\varepsilon } \quad &{}\text {in } \Omega \,,\\ \partial _\mathbf{n}u_{0,\varepsilon }^\lambda =0 \quad &{}\text {in } \partial \Omega \,, \end{array}\right. } \end{aligned}$$

is a possible choice. The existence of a unique approximated solution $(u_\varepsilon ^\lambda ,\mu _\varepsilon ^\lambda )$ for every $\lambda >0$ relies on a fixed-point argument, as in [24, Section 3.1]. For every $v\in L^2(0,T; W)$, since $W\hookrightarrow C^{0,\frac{1}{4}}(\overline{\Omega })$ by the Sobolev embeddings, thanks to the properties of $B_\varepsilon$ proved in Lemma 1 we have that $B_\varepsilon (v)\in L^2(0,T; H)$. Hence, by the classical literature on the local viscous Cahn–Hilliard equation (see again [18]), the map

$$\begin{aligned}&\Gamma _{\varepsilon }^{\lambda }: C^0([0,T]; H)\cap L^2(0,T; W) \rightarrow H^1(0,T; H)\cap L^\infty (0,T; V)\\&\quad \cap L^2(0,T; W)\,,\Gamma _\varepsilon ^\lambda :v\mapsto v_\varepsilon ^\lambda \,, \end{aligned}$$

is well-defined, where $(v_\varepsilon ^\lambda , w_\varepsilon ^\lambda )$ is the unique solution to the local viscous Cahn–Hilliard equation

$$\begin{aligned} \partial _t v_\varepsilon ^\lambda - \Delta w_\varepsilon ^\lambda = 0 \qquad&\text {in } Q\,,\\ w_\varepsilon ^\lambda = \tau _\varepsilon \partial _t v_\varepsilon ^\lambda -\lambda \Delta v_\varepsilon ^\lambda +\gamma _\lambda (v_\varepsilon ^\lambda ) +\Pi (v_\varepsilon ^\lambda )-\left( g_\varepsilon - B_\varepsilon (v)\right) \qquad&\text {in } Q\,,\\ \partial _\mathbf{n}u_\varepsilon ^\lambda = \partial _\mathbf{n}\mu _\varepsilon ^\lambda = 0 \qquad&\text {in } \Sigma \,,\\ v_\varepsilon ^\lambda (0)=u_{0,\varepsilon }^\lambda \qquad&\text {in } \Omega \,. \end{aligned}$$

Now, arguing as in [24, Section 3.1], exploiting the Lipschitz-continuity of $\gamma _\lambda$, the Sobolev embeddings, and the properties of $B_\varepsilon$ contained in Lemma 1, we deduce that there exist constants $L_\varepsilon ^\lambda >0$ and $\sigma >0$ such that, for every $v_1,v_2\in C^0([0,T]; H)\cap L^2(0,T; W)$, we have

$$\begin{aligned} \Vert \Gamma _\varepsilon ^\lambda (v_1) -\Gamma _\varepsilon ^\lambda (v_2)\Vert _{C^0([0,T]; H) \cap L^2(0,T; W)} \leqq L_\varepsilon ^\lambda T^\sigma \Vert v_1-v_2\Vert _{L^2(0,T; W)}\,. \end{aligned}$$

It follows that one can choose $T_0\in (0,T]$ sufficiently small so that $\Gamma _\varepsilon ^\lambda$ is a contraction on the respective functional spaces defined in $(0,T_0)$. Performing then a classical patching argument (we refer again to [24, Section 3.1] for details), we infer that $\Gamma _\varepsilon ^\lambda$ has a unique fixed point on the whole interval [0, *T*]. This proves that the approximated system (4.1)–(4.4) has a unique solution

$$\begin{aligned} u_\varepsilon ^\lambda \in H^1(0,T;H) \cap L^\infty (0,T; V)\cap L^2(0,T; W)\,,\qquad \mu _\varepsilon ^\lambda \in L^2(0,T;W)\,. \end{aligned}$$

### Uniform Estimates

We prove here some uniform estimates independently of $\lambda$ and $\varepsilon$. In what follows we will always assume that $\lambda \in [0,1]$. Moreover, $\varepsilon >0$ and $\tau _\varepsilon >0$ are still fixed.

We start by fixing $t\in [0,T]$, testing (4.1) with $\mu _\varepsilon ^{\lambda }$, (4.2) with $\partial _t u_\varepsilon ^{\lambda }$, taking the difference, and integrating the resulting equation on (0, *t*). We obtain

$$\begin{aligned}&\int _{Q_t}|\nabla \mu _\varepsilon ^\lambda (s,x)|^2\,\text {d}x \,\text {d}s +\tau _\varepsilon \int _{Q_t} |\partial _t u_\varepsilon ^\lambda (s,x)|^2\,\text {d}x \,\text {d}s\\&\qquad +\frac{\lambda }{2}\int _\Omega |\nabla u_\varepsilon ^\lambda (t,x)|^2\,\text {d}x + E_{\varepsilon }(u^\lambda _\varepsilon (t,\cdot )) + \int _\Omega (\hat{\gamma }_\lambda +\hat{\Pi })(u^\lambda _\varepsilon (t,x))\,\text {d}x \\&\quad \leqq \, \frac{\lambda }{2}\int _\Omega |\nabla u_{0,\varepsilon }^\lambda (x)|^2\,\text {d}x+E_{\varepsilon }(u_{0,\varepsilon }^\lambda )+ \int _\Omega (\hat{\gamma }_\lambda +\hat{\Pi })(u_{0,\varepsilon }^\lambda (x))\,\text {d}x \\&\qquad +\int _{Q_t} |g_\varepsilon (s,x)||\partial _t u(s,x)|\text {d}x\,\text {d}s. \end{aligned}$$

From the fact that

$$\begin{aligned} \int _\Omega \hat{\gamma }_{\lambda }(u_{0,\varepsilon }^\lambda (x))\,\text {d}x\leqq \int _\Omega \hat{\gamma }(u_{0,\varepsilon }^\lambda (x))\,\text {d}x\quad \text {for every}\,\lambda >0, \end{aligned}$$

using the uniform bound (4.6) as well as the Young inequality, we get

4.7 $$\begin{aligned}&\int _{Q_t} |\nabla \mu _\varepsilon ^\lambda (s,x)|^2\,\text {d}x\,\text {d}s +\frac{\tau _\varepsilon }{2}\int _{Q_t} |\partial _t u_\varepsilon ^\lambda (s,x)|^2\,\text {d}x\,\text {d}s \nonumber \\&\qquad + E_{\varepsilon }(u_\varepsilon ^\lambda (t,\cdot )) +\frac{\lambda }{2}\int _\Omega |\nabla u_\varepsilon ^\lambda (t,x)|^2\,\text {d}x \nonumber \\&\quad \leqq C_\varepsilon +\frac{\tau _\varepsilon }{4} \int _{Q_t} |\partial _t u^\lambda _\varepsilon (t,x)|^2\, \text {d}x\,\text {d}t + \frac{1}{\tau _\varepsilon }\int _0^T \int _{\Omega }|g_\varepsilon (t,x)|^2\,\text {d}x\, \text {d}t \end{aligned}$$

for every $t\in [0,T]$, where $C_\varepsilon >0$ is a constant independent of $\lambda$ and depending only on the initial datum $u_{0,\varepsilon }$.

From the arbitrariness of $t\in [0,T]$ we deduce that, for every $\lambda \in (0,1)$,

4.8 $$\begin{aligned}&\Vert \nabla \mu _\varepsilon ^\lambda \Vert _{L^2(0,T;H)} \leqq C_\varepsilon \,, \end{aligned}$$

4.9 $$\begin{aligned}&\Vert u_\varepsilon ^\lambda \Vert _{L^\infty (0,T;V_\varepsilon )\,}+ \Vert u_\varepsilon ^\lambda \Vert _{H^1(0,T;H)}+\lambda ^{1/2}\Vert \nabla u_\varepsilon ^\lambda \Vert _{L^\infty (0,T;H)} \leqq C_\varepsilon \,, \end{aligned}$$

hence, in addition by comparison in (4.1),

4.10 $$\begin{aligned} \Vert \Delta \mu _\varepsilon ^\lambda \Vert _{L^2(0,T; H)}\leqq C_\varepsilon \,. \end{aligned}$$

Furthermore, noting that $(u_\varepsilon ^\lambda )_\Omega = (u_{0,\varepsilon }^\lambda )_\Omega =(u_{0,\varepsilon })_\Omega$, we test (4.1) by $\mathcal N(u_\varepsilon ^\lambda - (u_{0,\varepsilon })_\Omega )$, (4.2) by $u_\varepsilon ^\lambda - (u_{0,\varepsilon })_\Omega$, and sum: we obtain, for almost every $t\in (0,T)$,

$$\begin{aligned}&\langle \partial _t u_\varepsilon ^\lambda (t), \mathcal N(u_\varepsilon ^\lambda (t) - (u_{0,\varepsilon })_\Omega )\rangle _{V} +\tau _\varepsilon \langle \partial _t u_\varepsilon ^\lambda (t), u_\varepsilon ^\lambda (t) - (u_{0,\varepsilon })_\Omega \rangle _V\\&\qquad +\lambda \int _\Omega |\nabla u_\varepsilon ^\lambda (t,x)|^2\text {d}x\\&\qquad +\int _\Omega B_\varepsilon (u_\varepsilon ^\lambda )(t,x)u_\varepsilon ^\lambda (t,x)\,\text {d}x +\int _\Omega \gamma _\lambda (u_\lambda (t,x)) (u_\varepsilon ^\lambda (t,x) - (u_{0,\varepsilon })_\Omega )\,\text {d}x\\&\quad =\int _\Omega \left( g^\varepsilon (t,x) - \Pi (u_\varepsilon ^\lambda )(t,x)\right) (u_\varepsilon ^\lambda (t,x) - (u_{0,\varepsilon })_\Omega )\,\text {d}x\,, \end{aligned}$$

where we have used that $\int _\Omega B_\varepsilon (u_\varepsilon ^\lambda (t,x))\,\text {d}x =0$ by the symmetry of the kernel $K_\varepsilon$. A classical argument shows that since $(u_{0,\varepsilon })_\Omega \in {\text {Int}}D(\gamma )$, then there are two constants $c_\varepsilon , c_\varepsilon '$, only depending on the position of $(u_{0,\varepsilon })_\Omega$, such that

$$\begin{aligned}&\Vert \gamma _\lambda (u_\varepsilon ^\lambda (t,\cdot )) \Vert _{L^1(\Omega )}\leqq c_\varepsilon \int _\Omega \gamma _\lambda (u_\varepsilon ^\lambda (t,x)) (u_\varepsilon ^\lambda (t,x)-(u_{0,\varepsilon })_\Omega ) \,\text {d}x +c_\varepsilon '\,,\\&\quad \text {for a.e. }t\in (0,T)\,. \end{aligned}$$

Arguing as in [24, Subsection 3.2], the estimates above and (4.8)–(4.9) yield then a control on $\Vert \gamma _\lambda (u_\lambda )\Vert _{L^2(0,T; L^1(\Omega ))}$. In particular, by comparison in (4.2) we get an estimate on $(\mu _\varepsilon ^\lambda )_\Omega$ in $L^2(0,T)$. Taking (4.8) and (4.10) into account, we deduce then that

4.11 $$\begin{aligned} \Vert \mu _\varepsilon ^\lambda \Vert _{L^2(0,T;W)}&\leqq C_\varepsilon \,. \end{aligned}$$

By comparison in (4.2) we infer that

$$\begin{aligned} \Vert -\lambda \Delta u_\varepsilon ^\lambda +B_\varepsilon (u_\varepsilon ^\lambda ) + \gamma _\lambda (u_\varepsilon ^\lambda )\Vert _{L^2(0,T; H)}\leqq C_\varepsilon \,. \end{aligned}$$

Testing $-\lambda \Delta u_\varepsilon ^\lambda +B_\varepsilon (u_\varepsilon ^\lambda ) + \gamma _\lambda (u_\varepsilon ^\lambda )$ by $\gamma _\lambda (u_\varepsilon ^\lambda )$ and noting that, by monotonicity of $\gamma _\lambda$,

$$\begin{aligned}&\int _\Omega (-\lambda \Delta u_\varepsilon ^\lambda (t,x) +B_\varepsilon (u_\varepsilon ^\lambda )(t,x)) \gamma _\lambda (u_\varepsilon ^\lambda )(t,x)\,\text {d}x\\&\quad =\lambda \int _\Omega \gamma _\lambda '(u_\varepsilon ^\lambda ) |\nabla u_\varepsilon ^\lambda (t,x)|^2\,\text {d}x\\&\qquad +\frac{1}{2}\int _\Omega \int _\Omega K_\varepsilon (x,y)\left( \gamma _\lambda (u_\varepsilon ^\lambda (t,x)) -\gamma _\lambda (u_\varepsilon ^\lambda (t,y))\right) \left( u_\varepsilon ^\lambda (t,x)-u_\varepsilon ^\lambda (t,y)\right) \,\text {d}x\,\text {d}y \geqq 0\,, \end{aligned}$$

by the estimate above and the Young inequality we also deduce that

4.12 $$\begin{aligned} \Vert -\lambda \Delta u_\varepsilon ^\lambda +B_\varepsilon (u_\varepsilon ^\lambda )\Vert _{L^2(0,T; H)} + \Vert \gamma _\lambda (u_\varepsilon ^\lambda )\Vert _{L^2(0,T; H)}\leqq C_\varepsilon \,. \end{aligned}$$

### Passage to the Limit as $\lambda \searrow 0$

In this section we analyze the passage to the limit as $\lambda \searrow 0$, with $\varepsilon >0$ and $\tau _\varepsilon >0$ still fixed. In view of the uniform bounds (4.8)–(4.12) and the Aubin-Lions lemma, up to the extraction of (not relabeled) subsequences we have the following convergences:

4.13 $$\begin{aligned} u_{\varepsilon }^\lambda&\rightarrow u_\varepsilon \quad&\text {in } C^0([0,T];V^*)\,,\end{aligned}$$

4.14 $$\begin{aligned} u_\varepsilon ^\lambda&{\mathop {\rightharpoonup }\limits ^{*}}u_\varepsilon&\text {in } L^\infty (0,T;V_\varepsilon )\cap H^1(0,T;H)\,, \end{aligned}$$

4.15 $$\begin{aligned} \lambda u_\varepsilon ^\lambda&\rightarrow 0&\text {in } L^\infty (0,T; V)\,, \end{aligned}$$

4.16 $$\begin{aligned} \mu _\varepsilon ^\lambda&\rightharpoonup \mu _\varepsilon&\text {in } L^2(0,T;W)\,,\end{aligned}$$

4.17 $$\begin{aligned} \gamma _\lambda (u^\lambda _\varepsilon )&\rightharpoonup \xi _{\varepsilon }&\text {in } L^2(0,T;H)\,, \end{aligned}$$

4.18 $$\begin{aligned} \Pi (u^\lambda _\varepsilon )&\rightharpoonup \Xi _{\varepsilon }&\text {in } L^2(0,T;H)\,, \end{aligned}$$

4.19 $$\begin{aligned} -\lambda \Delta u_\varepsilon ^\lambda + B_\varepsilon (u_\varepsilon ^\lambda )&\rightharpoonup \eta _\varepsilon&\text {in } L^2(0,T; H)\,, \end{aligned}$$

for some

$$\begin{aligned}\begin{gathered} u_\varepsilon \in H^1(0,T;H)\cap L^\infty (0,T;V_\varepsilon )\,,\qquad \mu _\varepsilon \in L^2(0,T;W)\,, \\ \xi _{\varepsilon } \in L^2(0,T;H)\,, \qquad \Xi _\varepsilon \in L^2(0,T; H)\,, \qquad \eta _\varepsilon \in L^2(0,T; H)\,. \end{gathered}\end{aligned}$$

From (4.14) and the fact that $B_\varepsilon \in \mathscr {L} (V_\varepsilon , V_\varepsilon ^*)$, it is readily seen that

$$\begin{aligned} B_\varepsilon (u_\varepsilon ^\lambda ){\mathop {\rightharpoonup }\limits ^{*}}B_\varepsilon (u_\varepsilon ) \qquad \text {in } L^\infty (0,T; V_\varepsilon ^*)\,. \end{aligned}$$

Moreover, from (4.15) and (4.19), it follows by comparison that

$$\begin{aligned} B_\varepsilon (u_\varepsilon ^\lambda )\rightharpoonup \eta _\varepsilon \qquad \text {in } L^2(0,T; V^*)\,. \end{aligned}$$

We deduce in particular that $B_\varepsilon (u_\varepsilon )=\eta _\varepsilon \in L^2(0,T; H)$, so that also $u_\varepsilon \in L^2(0,T; W_\varepsilon )$. The strong convergence (4.13) implies also that $u_\varepsilon (0)=u_{0,\varepsilon }$.

Passing to the limit in (4.1)-(4.4) in the weak topology of $L^2(0,T;H)$, we obtain

4.20 $$\begin{aligned} \partial _t u_\varepsilon - \Delta \mu _\varepsilon = 0 \quad&\text {in } L^2(0,T; H)\,,\end{aligned}$$

4.21 $$\begin{aligned} \mu _\varepsilon = \tau _\varepsilon \partial _t u_\varepsilon +{B}_\varepsilon (u_\varepsilon ) +\xi _{\varepsilon } +\Xi _{\varepsilon }-g_\varepsilon \quad&\text {in } L^2(0,T; H)\,,\end{aligned}$$

4.22 $$\begin{aligned} \partial _\mathbf{n}\mu _\varepsilon = 0 \quad&\text {in } L^2(\Sigma )\,,\end{aligned}$$

4.23 $$\begin{aligned} u_\varepsilon (0)=u_{0,\varepsilon } \quad&\text {in } H\,. \end{aligned}$$

We proceed now providing an identification of the nonlinear terms $\xi _\varepsilon$ and $\Xi _\varepsilon$: we adapt an argument performed in [22, Subsection 3.6]. To this end, since $\Pi$ is Lipschitz-continuous, there exists $\alpha >0$ such that the operator

$$\begin{aligned} \gamma +\Pi +\alpha \tau _\varepsilon \,\mathrm {Id}:\mathbb {R}\rightarrow 2^{\mathbb {R}} \end{aligned}$$

is maximal monotone. For example, one can choose $\alpha :=\frac{2}{\tau _\varepsilon }\Vert \Pi '\Vert _{L^\infty (\mathbb R)}$ (recall that $\tau _\varepsilon >0$ is fixed). Multiplying (4.2) by $e^{-\alpha t}$, we obtain

$$\begin{aligned} e^{-\alpha t}\mu _{\varepsilon }^\lambda= & {} \tau _\varepsilon \partial _t (e^{-\alpha t}u_\varepsilon ^\lambda )-\lambda \Delta (e^{-\alpha t}u_\varepsilon ^\lambda )+B_\varepsilon (e^{-\alpha t}u_\varepsilon ^\lambda ) +e^{-\alpha t} (\gamma _\lambda (u_\varepsilon ^\lambda )\\&+\Pi (u_\varepsilon ^\lambda )+\alpha \tau _\varepsilon u_\varepsilon ^\lambda - g_\varepsilon ). \end{aligned}$$

Thus, testing the previous equation by $e^{-\alpha t}u_\varepsilon ^\lambda$ and integrating in time yields

$$\begin{aligned}&\limsup _{\lambda \rightarrow 0}\int _Q e^{-2\alpha s}(\gamma _\lambda (u_\varepsilon ^\lambda (s,x)) +\Pi (u_\varepsilon ^\lambda (s,x)) +\alpha \tau _\varepsilon u_\varepsilon ^\lambda (s,x))u_\varepsilon ^\lambda (s,x) \, \text {d}x\,\text {d}s\\&\quad \leqq \limsup _{\lambda \rightarrow 0}\left[ \int _Q e^{-2\alpha s}\mu _\varepsilon ^\lambda (s,x) u_\varepsilon ^\lambda (s,x)\,\text {d}x\,\text {d}s -\lambda \int _Q e^{-2\alpha s} |\nabla u_\varepsilon ^\lambda (s,x)|^2\,\text {d}x\,\text {d}s\right. \\&\qquad \qquad \left. -\frac{\tau _\varepsilon }{2}\int _\Omega e^{-2\alpha T}|u_\varepsilon ^\lambda (T,x)|^2\,\text {d}x +\frac{\tau _\varepsilon }{2}\int _\Omega |u_{0,\varepsilon }^\lambda (x)|^2\,\text {d}x\right. \\&\qquad \left. -2\int _0^T e^{-2\alpha s}E_\varepsilon (u_\varepsilon ^\lambda (s,\cdot ))\,\text {d}s\right. \\&\qquad \qquad \left. +\int _Q e^{-2\alpha s} g_\varepsilon (s,x)u_\varepsilon ^\lambda (s,x)\,\text {d}x\,\text {d}s\right] . \end{aligned}$$

On the one hand, owing to (4.13) and (4.16),

$$\begin{aligned}&\lim _{\lambda \rightarrow 0}\int _Q e^{-2\alpha s} (\mu _\varepsilon ^\lambda (s,x)+g_\varepsilon (s,x)) u_\varepsilon ^\lambda (s,x)\,\text {d}x\,\text {d}s\\&\quad =\int _Q e^{-2\alpha s}(\mu _\varepsilon (s,x)+g_\varepsilon (s,x)) u_\varepsilon (s,x)\,\text {d}x\,\text {d}s\,. \end{aligned}$$

On the other hand, by the weak lower semicontinuity of the norms, the convergence (4.14), and the assumption (4.5), we have

$$\begin{aligned}&\limsup _{\lambda \rightarrow 0} \left[ -\lambda \int _Q e^{-2\alpha s}|\nabla u_\varepsilon ^\lambda (s,x)|^2\,\text {d}x\,\text {d}s\right. \\&\qquad \qquad \left. -\frac{\tau _\varepsilon }{2}\int _\Omega e^{-2\alpha T}|u_\varepsilon ^\lambda (T,x)|^2\,\text {d}x +\frac{\tau _\varepsilon }{2}\int _\Omega |u_{0,\varepsilon }^\lambda (x)|^2\,\text {d}x\right. \\&\qquad \left. -2\int _0^T e^{-2\alpha s} E_\varepsilon (u_\varepsilon ^\lambda (s,\cdot ))\,\text {d}s\right] \\&\quad \leqq -\frac{\tau _\varepsilon }{2}\liminf _{\lambda \rightarrow 0} \int _\Omega e^{-2\alpha T}|u_\varepsilon ^\lambda (t,x)|^2\,\text {d}x +\frac{\tau _\varepsilon }{2}\limsup _{\lambda \rightarrow 0} \int _\Omega |u_{0,\varepsilon }^\lambda (x)|^2\,\text {d}x\\&\qquad -2\liminf _{\lambda \rightarrow 0} \int _0^T e^{-2\alpha s} E_\varepsilon (u_\varepsilon ^\lambda (s,\cdot ))\,\text {d}s\\&\quad \leqq -\frac{\tau _\varepsilon }{2}\int _\Omega e^{-2\alpha T}|u_\varepsilon (T,x)|^2\,\text {d}x +\frac{\tau _\varepsilon }{2}\int _\Omega |u_{0,\varepsilon }(x)|^2\,\text {d}x\\&\qquad -2\int _0^T e^{-2\alpha s}E_\varepsilon (u_\varepsilon (s,\cdot ))\,\text {d}s\,. \end{aligned}$$

Hence, we deduce that

4.24 $$\begin{aligned}&\limsup _{\lambda \rightarrow 0}\int _Q e^{-2\alpha s} (\gamma _\lambda (u_\varepsilon ^\lambda (s,x))+ \Pi (u_\varepsilon ^\lambda (s,x))+ \alpha \tau _\varepsilon u_\varepsilon ^\lambda (s,x))u_\varepsilon ^\lambda (s,x) \, \text {d}x\,\text {d}s \nonumber \\&\quad \leqq \int _Q e^{-2\alpha s}(\mu _\varepsilon (s,x)+g_\varepsilon (s,x))u_\varepsilon (s,x)\,\text {d}x\,\text {d}s \nonumber \\&\qquad -\frac{\tau _\varepsilon }{2}\int _\Omega e^{-2\alpha T}|u_\varepsilon (T,x)|^2\,\text {d}x +\frac{\tau _\varepsilon }{2}\int _\Omega |u_{0,\varepsilon }(x)|^2\,\text {d}x \nonumber \\&\qquad -2\int _0^T e^{-2\alpha s}E_\varepsilon (u_\varepsilon (s,\cdot ))\,\text {d}s\,. \end{aligned}$$

Testing (4.21) by $e^{-2\alpha t}u_\varepsilon$ and integrating in time, the right-hand side of (4.24) rewrites as

$$\begin{aligned}&\limsup _{\lambda \rightarrow 0}\int _Q e^{-2\alpha s} (\gamma _\lambda (u_\varepsilon ^\lambda (s,x)) +\Pi (u_\varepsilon ^\lambda (s,x))+\alpha \tau _\varepsilon u_\varepsilon ^\lambda (s,x))u_\varepsilon ^\lambda (s,x) \text {d}x\,\text {d}s\\&\quad \leqq \int _0^t\int _\Omega e^{-2\alpha s}(\xi _{\varepsilon }(s,x)+\Xi _{\varepsilon }(s,x)+ \alpha \tau _\varepsilon u_{\varepsilon }(s,x))u_{\varepsilon }(s,x)\,\text {d}x\,\text {d}s\,. \end{aligned}$$

Since the bilinear form

$$\begin{aligned} (v_1,v_2)\mapsto \int _Qe^{-2\alpha x} v_1(s,x)v_2(s,x)\,\text {d}x\,\text {d}s\,, \quad v_1,v_2\in L^2(Q)\,, \end{aligned}$$

is an equivalent scalar product on $L^2(Q)$, by the maximal monotonicity of $\gamma +\Pi +\alpha \tau _\varepsilon \,\mathrm {Id}$ we conclude that

4.25 $$\begin{aligned} \xi _{\varepsilon }+\Xi _{\varepsilon }+\alpha \tau _\varepsilon u_{\varepsilon }\in (\gamma +\Pi +\alpha \tau _\varepsilon \,\mathrm {Id})(u_\varepsilon ) \quad \text {a.e. in } Q\,. \end{aligned}$$

This allows us to show the further strong convergences

4.26 $$\begin{aligned} u_\varepsilon ^\lambda (t) \rightarrow u_\varepsilon (t) \quad \text {in } H \quad \forall \,t\in [0,T]\,, \qquad u_\varepsilon ^\lambda \rightarrow u_\varepsilon \quad \text {in } L^2(0,T; V_\varepsilon )\,. \end{aligned}$$

Indeed, taking the difference between (4.2) and (4.21), multiplying again by $e^{-\alpha t}$, and testing by $e^{-\alpha t}(u_\varepsilon ^\lambda -u_\varepsilon )$, we get

$$\begin{aligned}&\frac{\tau _\varepsilon }{2}\int _\Omega e^{-2\alpha t} |(u_\varepsilon ^\lambda -u_\varepsilon )(t,x)|^2\,\text {d}x +\lambda \int _{Q_t}e^{-2\alpha s}|\nabla u_\varepsilon ^\lambda (s,x)|^2\,\text {d}x\,\text {d}s\\&\qquad +2\int _0^Te^{-2\alpha s} E_\varepsilon ((u_\varepsilon ^\lambda -u_\varepsilon )(s,x))\,\text {d}s\\&\qquad +\int _{Q_t}e^{-2\alpha s} \left( \gamma _\lambda (u_\varepsilon ^\lambda ) +\Pi (u_\varepsilon ^\lambda ) +\alpha \tau _\varepsilon u_{\varepsilon }^\lambda -(\xi _\varepsilon + \Xi _\varepsilon + \alpha \tau _\varepsilon u_\varepsilon ) \right) (s,x) (u_\varepsilon ^\lambda -u_\varepsilon ) (s,x)\,\text {d}x\,\text {d}s\\&\quad =\frac{\tau _\varepsilon }{2}\int _\Omega |u_{0,\varepsilon }^\lambda (x)- u_{0,\varepsilon }(x)|^2\,\text {d}x +\int _{Q_t}e^{-2\alpha s} (\mu _\lambda -\mu )(s,x)(u_\varepsilon ^\lambda -u_\varepsilon )(s,x) \,\text {d}x\,\text {d}s\\&\qquad -\lambda \int _{Q_t}e^{-2\alpha s} \Delta u_\varepsilon ^\lambda (s,x)u_\varepsilon (s,x) \,\text {d}x\,\text {d}s\,. \end{aligned}$$

We use now the notation $J^\gamma _\lambda := (\mathrm {Id}+\lambda \gamma )^{-1}:\mathbb {R}\rightarrow \mathbb {R}$ for the resolvent of $\gamma$. Summing and subtracting $J_\lambda ^\gamma (u_\varepsilon ^\lambda )$ in the last term on the left-hand side, rearranging the terms, and recalling that $u_\varepsilon ^\lambda -J_\lambda ^\gamma (u_\varepsilon ^\lambda )= \lambda \gamma _\lambda (u_\varepsilon ^\lambda )$, we infer that, for every $t\in [0,T]$,

$$\begin{aligned}&\frac{\tau _\varepsilon }{2}\int _\Omega e^{-2\alpha t} |(u_\varepsilon ^\lambda -u_\varepsilon )(t,x)|^2\,\text {d}x +2\int _0^Te^{-2\alpha s} E_\varepsilon ((u_\varepsilon ^\lambda -u_\varepsilon )(s,x))\,\text {d}s\\&\qquad +\int _{Q_t}e^{-2\alpha s} \left( \gamma _\lambda (u_\varepsilon ^\lambda ) {+}\Pi (J^\gamma _\lambda (u_\varepsilon ^\lambda ))\right. \\&\qquad \left. +\alpha \tau _\varepsilon J^\gamma _\lambda (u_{\varepsilon }^\lambda ) {-}(\xi _\varepsilon {+} \Xi _\varepsilon {+} \alpha \tau _\varepsilon u_\varepsilon ) \right) (s,x) (J_\lambda (u_\varepsilon ^\lambda ){-}u_\varepsilon )(s,x)\,\text {d}x\,\text {d}s\\&\quad \leqq \frac{\tau _\varepsilon }{2}\int _\Omega |u_{0,\varepsilon }^\lambda (x)- u_{0,\varepsilon }(x)|^2\,\text {d}x + \int _{Q_t}e^{-2\alpha s} (\mu _\lambda -\mu )(s,x)(u_\varepsilon ^\lambda -u_\varepsilon )(s,x) \,\text {d}x\,\text {d}s\\&\qquad -\int _{Q_t}e^{-2\alpha s} B_\varepsilon (u_\varepsilon ^\lambda (s,x)))u_\varepsilon (s,x) \,\text {d}x\,\text {d}s\\&\qquad +\int _{Q_t}e^{-2\alpha s} (-\lambda \Delta u_\varepsilon ^\lambda (s,x) +B_\varepsilon (u_\varepsilon ^\lambda (s,x)))u_\varepsilon (s,x) \,\text {d}x\,\text {d}s\\&\qquad +\int _{Q_t}e^{-2\alpha s} \left( \Pi (J^\gamma _\lambda (u_\varepsilon ^\lambda (s,x))) {-} \Pi (u_\varepsilon ^\lambda (s,x))\right. \\&\qquad \left. {+}\alpha \tau _\varepsilon (J^\gamma _\lambda (u_\varepsilon ^\lambda ){-}u_\varepsilon ^\lambda )(s,x)\right) (J_\lambda (u_\varepsilon ^\lambda ){-}u_\varepsilon ) (s,x)\,\text {d}x\,\text {d}s\\&\qquad -\lambda \int _{Q_t}e^{-2\alpha s} \left( \gamma _\lambda (u_\varepsilon ^\lambda ) +\Pi (u_\varepsilon ^\lambda ) +\alpha \tau _\varepsilon u_{\varepsilon }^\lambda -(\xi _\varepsilon + \Xi _\varepsilon + \alpha \tau _\varepsilon u_\varepsilon ) \right) \\&\quad (s,x) \gamma _\lambda (u_\varepsilon ^\lambda (s,x))\,\text {d}x\,\text {d}s \,. \end{aligned}$$

Recalling that $\gamma _\lambda (r)\in \gamma (J_\lambda ^\gamma (r))$ for every $r\in \mathbb {R}$, by (4.25) and the monotonicity of the operator $\gamma + \Pi + \alpha \tau _\varepsilon \mathrm {Id}$, the third term on the left-hand side is nonnegative. Let us show that the right-hand side converges to 0, analyzing each term separately. The first two terms on the right-hand side converge to 0 thanks to (4.5), (4.13) and (4.16). Moreover, thanks to (4.14), (4.19), and the fact that $u_\varepsilon \in L^2(0,T; W_\varepsilon )$, we have

$$\begin{aligned} -\int _{Q_t}e^{-2\alpha s} B_\varepsilon (u_\varepsilon ^\lambda (s,x))u_\varepsilon (s,x) \,\text {d}x\,\text {d}s \rightarrow -\int _{Q_t}e^{-2\alpha s} B_\varepsilon (u_\varepsilon (s,x))u_\varepsilon (s,x) \,\text {d}x\,\text {d}s \end{aligned}$$

and

$$\begin{aligned}&\int _{Q_t}e^{-2\alpha s} (-\lambda \Delta u_\varepsilon ^\lambda (s,x) +B_\varepsilon (u_\varepsilon ^\lambda (s,x)))u_\varepsilon (s,x) \,\text {d}x\,\text {d}s\\&\quad \rightarrow \int _{Q_t}e^{-2\alpha s} B_\varepsilon (u_\varepsilon (s,x))u_\varepsilon (s,x) \,\text {d}x\,\text {d}s\,. \end{aligned}$$

Finally, since $(\gamma _\lambda (u_\varepsilon ^\lambda ))_\lambda$ is bounded in $L^2(0,T; H)$ by (4.12), using the Lipschitz-continuity of *Pi*, the last two terms on the right-hand side can be handled by

$$\begin{aligned}&\lambda \Vert \gamma _\lambda (u_\varepsilon ^\lambda )\Vert _{L^2(0,T; H)} \left( \Vert J_\lambda ^\gamma (u_\varepsilon ^\lambda )\Vert _{L^2(0,T; H)} +\Vert u_\varepsilon \Vert _{L^2(0,T; H)}\Vert \right. \\&\qquad \left. +\Vert \gamma _\lambda (u_\varepsilon ^\lambda ) +\Pi (u_\varepsilon ^\lambda ) +\alpha \tau _\varepsilon u_{\varepsilon }^\lambda -(\xi _\varepsilon + \Xi _\varepsilon + \alpha \tau _\varepsilon u_\varepsilon )\Vert _{L^2(0,T; H)} \right) \leqq C_\varepsilon \lambda \rightarrow 0\,. \end{aligned}$$

Since $t\in [0,T]$ is arbitrary, the strong convergences (4.26) follows. In particular, this readily implies that $\Xi _\varepsilon =\Pi (u_\varepsilon )$ and $\xi _\varepsilon \in \gamma (u_\varepsilon )$ almost everywhere in *Q* by the Lipschitz-continuity of $\Pi$ and by the maximal monotonicity of $\gamma$, respectively.

It is then clear that $(u_\varepsilon ,\mu _\varepsilon ,\xi _\varepsilon )$ is a solution to the nonlocal viscous Cahn–Hilliard equation in the sense of (3.11)–(3.16). This completes the proof of the first assertion of Theorem 3.2.

### Continuous Dependence

Let $(u_{0,\varepsilon }^1, g_\varepsilon ^1)$ and $(u_{0,\varepsilon }^2, g_\varepsilon ^2)$ satisfy the assumptions (3.9)–(3.10) with $(u_{0,\varepsilon }^1)_\Omega = (u_{0,\varepsilon }^2)_\Omega$, and let $(u_\varepsilon ^1, \mu _\varepsilon ^1,\xi _\varepsilon ^1)$ and $(u_\varepsilon ^2, \mu _\varepsilon ^2, \xi _\varepsilon ^2)$ be any corresponding solutions to (3.11)–(3.16).

We observe that their difference solves

$$\begin{aligned} \partial _t(u_\varepsilon ^1-u_\varepsilon ^2)-\Delta (\mu _\varepsilon ^1-\mu _\varepsilon ^2)=0 \quad&\text {in } Q\,,\\ \mu _\varepsilon ^1-\mu _\varepsilon ^2=\tau _\varepsilon \partial _t (u_\varepsilon ^1-u_\varepsilon ^2)+B_\varepsilon (u_\varepsilon ^1-u_\varepsilon ^2) +\xi _\varepsilon ^1-\xi _\varepsilon ^2 + \Pi (u_\varepsilon ^1)-\Pi (u_\varepsilon ^2) -(g_\varepsilon ^1-g_\varepsilon ^2) \quad&\text {in Q}\,,\\ \partial _\mathbf{n}(\mu _\varepsilon ^1-\mu _\varepsilon ^2)=0 \quad&\text {in }\Sigma \,,\\ (u_\varepsilon ^1-u_\varepsilon ^2)(0)=0 \quad&\text {in } \Omega \,. \end{aligned}$$

By the assumption on the initial data, we have that $(u_\varepsilon ^1-u_\varepsilon ^2)_\Omega =0$. Therefore, we can test the first equation by $\mathcal N(u_\varepsilon ^1-u_\varepsilon ^2)$, the second by $u_\varepsilon ^1-u_\varepsilon ^2$, and take the difference: by performing classical computations we get

$$\begin{aligned}&\frac{1}{2}\Vert (u_\varepsilon ^1-u_\varepsilon ^2)(t)\Vert _{V^*}^2+\frac{\tau _\varepsilon }{2}\Vert (u_\varepsilon ^1-u_\varepsilon ^2)(t)\Vert _{H}^2 +2\int _0^t E_\varepsilon (u_\varepsilon ^1-u_\varepsilon ^2)(s)\,\text {d}s\\&\qquad +\int _{Q_t} (\xi _\varepsilon ^1-\xi _\varepsilon ^2)(s,x)(u_\varepsilon ^1-u_\varepsilon ^2)(s,x) \,\text {d}x\,\text {d}s\\&\quad =\frac{1}{2}\Vert (u_{0,\varepsilon }^1 -u_{0,\varepsilon }^2)\Vert _{V^*}^2+\frac{\tau _\varepsilon }{2}\Vert (u_{0,\varepsilon }^1 -u_{0,\varepsilon }^2)\Vert _{H}^2\\&\qquad +\int _{Q_t}\left( g_\varepsilon ^1-g_\varepsilon ^2 -\Pi (u_\varepsilon ^1)+\Pi (u_\varepsilon ^2)\right) (s,x) (u_\varepsilon ^1-u_\varepsilon ^2)(s,x)\,. \end{aligned}$$

The last term on the left-hand side is nonnegative by the monotonicity of $\gamma$. Hence, the continuous-dependence property stated in Theorem 3.2 follows from the Lipschitz-continuity of $\Pi$ and the Gronwall lemma.

## Proof of Theorem 3.3

This section is devoted to study the asymptotic behavior of solutions to the nonlocal viscous Cahn–Hilliard equation as $\varepsilon \searrow 0$. Let us recall that the family of data $(u_{0,\varepsilon },g_\varepsilon )_{\varepsilon >0}$ are assumed to satisfy (3.17)–(3.20), while $(u_\varepsilon , \mu _\varepsilon , \xi _\varepsilon )$ is a corresponding solution to (3.11)–(3.16).

### The Case $\tau >0$

We consider here the case $\tau >0$, so that $\tau _\varepsilon \rightarrow \tau >0$. As a major consequence, this implies that it is not restrictive to assume that

5.1 $$\begin{aligned} \exists \,\tau _*>0: \quad \tau _\varepsilon \geqq \tau _* \quad \forall \,\varepsilon \in (0,\varepsilon _0)\,. \end{aligned}$$

We test (3.14) by $\mu _\varepsilon$, (3.15) by $\partial _t u_\varepsilon$, take the difference, and integrate on $Q_t$: recalling (3.18) and using the Young inequality, we deduce that

$$\begin{aligned}&\int _{Q_t}|\nabla \mu _\varepsilon (s,x)|^2\,\text {d}x \,\text {d}s +\tau _\varepsilon \int _{Q_t} |\partial _t u_\varepsilon (s,x)|^2\,\text {d}x \,\text {d}s + E_{\varepsilon }(u_\varepsilon (t,\cdot )) \\&\quad +\int _\Omega (\hat{\gamma }+\hat{\Pi }) (u_\varepsilon (t,x))\,\text {d}x \\&\quad \leqq E_{\varepsilon }(u_{0,\varepsilon })+ \int _\Omega (\hat{\gamma }+\hat{\Pi })(u_{0,\varepsilon }(x))\,\text {d}x +\frac{\tau _\varepsilon }{2}\int _{Q_t}|\partial _t u_\varepsilon (s,x)|^2\,\text {d}x\,\text {d}s\\&\qquad +\frac{1}{\tau _\varepsilon }\int _{Q_t}|g_\varepsilon (s,x)|^2\,\text {d}x\,\text {d}s\,. \end{aligned}$$

Note that $\frac{1}{\tau _\varepsilon }\leqq \frac{1}{\tau _*}$ by (5.1). Hence, rearranging the terms and using (3.17) we infer that there exists a constant $C>0$, independent of $\varepsilon$, such that

$$\begin{aligned} \Vert \nabla \mu _\varepsilon \Vert _{L^2(0,T; H)} + \Vert u_\varepsilon \Vert _{H^1(0,T; H)\cap L^\infty (0,T; V_\varepsilon )}\leqq C\, \end{aligned}$$

hence also, by comparison in (3.14),

$$\begin{aligned} \Vert \Delta \mu _\varepsilon \Vert _{L^2(0,T; H)}\leqq C\,. \end{aligned}$$

Now, we can proceed as in the previous Sect. 4.2. Since $(u_\varepsilon )_\Omega =(u_{0,\varepsilon })_\Omega$, we can test (3.14) by $\mathcal N(u_\varepsilon - (u_{0,\varepsilon })_\Omega )$, (3.15) by $u_\varepsilon - (u_{0,\varepsilon })_\Omega$, and sum: we obtain, for almost every $t\in (0,T)$,

$$\begin{aligned}&\langle \partial _t u_\varepsilon (t), \mathcal N(u_\varepsilon (t) - (u_{0,\varepsilon })_\Omega )\rangle _{V} +\tau _\varepsilon \langle \partial _t u_\varepsilon (t), u_\varepsilon (t) - (u_{0,\varepsilon })_\Omega \rangle _V +2E_\varepsilon (u_\varepsilon (t,x))\\&\qquad +\int _\Omega \xi _\varepsilon (t,x) (u_\varepsilon (t,x) - (u_{0,\varepsilon })_\Omega )\,\text {d}x\\&\quad =\int _\Omega \left( g^\varepsilon (t,x) - \Pi (u_\varepsilon )(t,x)\right) (u_\varepsilon (t,x) - (u_{0,\varepsilon })_\Omega )\,\text {d}x. \end{aligned}$$

Again, by the estimates already performed, all the terms are bounded in $L^2(0,T)$ except

$$\begin{aligned} \int _\Omega \xi _\varepsilon (t,x) (u_\varepsilon (t,x) - (u_{0,\varepsilon })_\Omega )\,\text {d}x\,. \end{aligned}$$

Thanks to assumption (3.19), there are two constants $c, c'>0$, independent of $\varepsilon$, such that

$$\begin{aligned} \Vert \xi _\varepsilon (t,\cdot )\Vert _{L^1(\Omega )}\leqq c\int _\Omega \xi _\varepsilon (t,\cdot ) (u_\varepsilon (t,x)-(u_{0,\varepsilon })_\Omega )\,\text {d}x +c'\,. \end{aligned}$$

Hence, we deduce that

$$\begin{aligned} \Vert \xi _\varepsilon \Vert _{L^2(0,T; L^1(\Omega ))}\leqq C\,, \end{aligned}$$

which implies, by comparison in (3.15), that

$$\begin{aligned} \Vert (\mu _\varepsilon )_\Omega \Vert _{L^2(0,T)}\leqq C\,. \end{aligned}$$

We deduce that

$$\begin{aligned} \Vert \mu _\varepsilon \Vert _{L^2(0,T; W)}\leqq C\,. \end{aligned}$$

Thus, by comparison in (3.15) and by monotonicity of $\gamma$, we obtain that

$$\begin{aligned} \Vert B_\varepsilon (u_\varepsilon )\Vert _{L^2(0,T; H)} + \Vert \xi _\varepsilon \Vert _{L^2(0,T; H)}\leqq C\,. \end{aligned}$$

By the Aubin-Lions compactness theorem we infer that, up to the extraction of (not relabeled) subsequences, as $\varepsilon \searrow 0$,

5.2 $$\begin{aligned} u_{\varepsilon }\rightarrow u \qquad&\text {in } C^0([0,T]; V^*)\,,\end{aligned}$$

5.3 $$\begin{aligned} u_\varepsilon \rightharpoonup u \qquad&\text {in } H^1(0,T;H)\,, \end{aligned}$$

5.4 $$\begin{aligned} B_\varepsilon (u_\varepsilon )\rightharpoonup \eta \qquad&\text {in } L^2(0,T;H)\,,\end{aligned}$$

5.5 $$\begin{aligned} \mu _\varepsilon \rightharpoonup \mu \qquad&\text {in } L^2(0,T;W)\,,\end{aligned}$$

5.6 $$\begin{aligned} \xi _\varepsilon \rightharpoonup \xi \qquad&\text {in } L^2(0,T;H) \end{aligned}$$

for some

$$\begin{aligned} u \in H^1(0,T; H)\,, \qquad \mu \in L^2(0,T; W)\,, \qquad \xi ,\eta \in L^2(0,T; H)\,. \end{aligned}$$

We proceed by showing the strong convergence

5.7 $$\begin{aligned} u_\varepsilon \rightarrow u \qquad \text {in } C^0([0,T]; H)\,. \end{aligned}$$

To this end, we show that the sequence $(u_\varepsilon )_\varepsilon$ is Cauchy in $C^0([0,T]; H)$. For any arbitrary $\sigma >0$, we apply Lemma 3 with the choice $\delta :=\frac{\sigma }{4C}$, where $C>0$ is the constant obtained in the estimates above. We deduce that there exists $\bar{\varepsilon }=\bar{\varepsilon }_\sigma$ and $C_\sigma >0$ such that

$$\begin{aligned} \Vert (u_{\varepsilon _1}-u_{\varepsilon _2})(t)\Vert _H^2\leqq \frac{\sigma }{4C}\left( E_{\varepsilon _1}(u_{\varepsilon _1}(t)) +E_{\varepsilon _2}(u_{\varepsilon _2}(t))\right) +C_\sigma \Vert (u_{\varepsilon _1}-u_{\varepsilon _2})(t)\Vert _{V^*}^2 \end{aligned}$$

for every $\varepsilon _1,\varepsilon _2\in (0,\bar{\varepsilon }_\sigma )$, for every $t\in [0,T]$. Thanks to (5.2), there exists $\tilde{\varepsilon }_\sigma \in (0,\bar{\varepsilon }_\sigma )$ such that

$$\begin{aligned} \Vert u_{\varepsilon _1}-u_{\varepsilon _2}\Vert ^2_{C^0([0,T];V^*)}\leqq \frac{\sigma }{2C_\sigma } \quad \forall \,\varepsilon _1,\varepsilon _2 \in (0,\tilde{\varepsilon }_\sigma )\,. \end{aligned}$$

Hence, taking the supremum in time and using the estimates above we infer that

$$\begin{aligned}&\Vert u_{\varepsilon _1}-u_{\varepsilon _2}\Vert _{C^0([0,T];H)}^2\\&\quad \leqq \frac{\sigma }{4C}\left( \Vert E_{\varepsilon _1}(u_{\varepsilon _1})\Vert _{L^\infty (0,T)}+ \Vert E_{\varepsilon _2}(u_{\varepsilon _2})\Vert _{L^\infty (0,T)}\right) +C_\sigma \Vert u_{\varepsilon _1}-u_{\varepsilon _2}\Vert ^2_{C^0([0,T];V^*)}\\&\quad \leqq \frac{\sigma }{4C}(C+C) + C_\sigma \frac{\sigma }{2C_\sigma } = \sigma \end{aligned}$$

for every $\varepsilon _1,\varepsilon _2\in (0,\tilde{\varepsilon }_\sigma )$. Since $\sigma >0$ is arbitrary, we obtain the strong convergence (5.7).

Now, from (5.7) and the Lipschitz continuity of $\Pi$, it follows that

$$\begin{aligned} \Pi (u_\varepsilon ) \rightarrow \Pi (u) \qquad \text {in } C^0([0,T]; H)\,, \end{aligned}$$

while the strong-weak closure of $\gamma$ readily ensures that $\xi _\varepsilon \in \gamma (u_\varepsilon )$ almost everywhere in *Q*.

To conclude the proof of the theorem, it remains to prove additional spatial regularity for *u* and to provide an identification of $\eta$. First of all, note that since $(u_\varepsilon )_\varepsilon$ is bounded in $L^\infty (0,T; V_\varepsilon )$, by the Ponce criterion [51, Theorem 1.2] we have that $u\in L^\infty (0,T; V)$.

Let us identify now the term $\eta$. We first observe that by Lemma 1 there holds $DE_\varepsilon =B_\varepsilon$ as operators on $V_\varepsilon$. Thus, by Lemma 2, and by the continuous inclusion of *V* into $V_\varepsilon$, we deduce

$$\begin{aligned} E_\varepsilon (z_1)+ \langle B_\varepsilon (z_1),z_2-z_1\rangle _{V_\varepsilon ^*,V_\varepsilon }\leqq E_\varepsilon (z_2) \quad \forall \, z_1,z_2\in V\,. \end{aligned}$$

Hence, for all $z\in L^2(0,T; V)$ we deduce that

5.8 $$\begin{aligned}&\int _0^TE_\varepsilon (u_\varepsilon (t,\cdot ))\, \text {d}t +\int _0^T\int _\Omega B_\varepsilon (u_\varepsilon (t,x)) (z(t,x)-u_\varepsilon (t,x))\, \text {d}x \, \text {d}t \nonumber \\&\quad \leqq \int _0^T E_\varepsilon (z(t,\cdot ))\, \text {d}t. \end{aligned}$$

Owing to Lemma 2, and to the dominated convergence theorem, we have

$$\begin{aligned} \int _0^T E_\varepsilon (z(t,\cdot ))\, \text {d}t\rightarrow \frac{1}{2}\int _0^T\int _\Omega |\nabla z(x,t)|^2\text {d}x\,\text {d}t\,. \end{aligned}$$

On the one hand, (5.4) and (5.7) yield

$$\begin{aligned}&\int _0^T\int _\Omega B_\varepsilon (u_\varepsilon (t,x)) (z(t,x)-u_\varepsilon (t,x))\, \text {d}x\, \text {d}t \\&\quad \rightarrow \int _0^T\int _\Omega \eta (t,x)(z(t,x)-u(t,x))\, \text {d}x\, \text {d}t. \end{aligned}$$

On the other hand, by the Gamma-convergence result in Lemma 2 and by Fatou’s Lemma,

$$\begin{aligned} \liminf _{\varepsilon \rightarrow 0}\int _0^T E_\varepsilon (u_\varepsilon (t,\cdot ))\,\text {d}t\geqq \frac{1}{2}\int _Q|\nabla u(t,x)|^2\,\text {d}x\,\text {d}t. \end{aligned}$$

Letting $\varepsilon \rightarrow 0$ in (5.8) and recalling that $u\in L^\infty (0,T; V)$, we obtain the inequality

5.9 $$\begin{aligned} \frac{1}{2}\int _Q |\nabla u(t,x)|^2\,\text {d}x\, \text {d}t +\int _Q \!\eta (t,x) (z(t,x)-u(t,x))\, \text {d}x\, \text {d}t \leqq \frac{1}{2}\int _Q|\nabla z(t,x)|^2\,\text {d}x\, \text {d}t \end{aligned}$$

for every $z\in L^2(0,T; V)$, which in turn implies that $-\Delta u=\eta \in L^2(0,T;H)$. Since $u\in L^\infty (0,T; V)$ and $\Delta u \in L^2(0,T; H)$ in the sense of distributions for example, by [41, Thm. 2.27] the normal derivative $\partial _\mathbf{n} u \in L^2(0,T; H^{-1/2}(\partial \Omega ))$ is well defined. We infer that, for almost every $t\in (0,T)$ and for every $\varphi \in V$,

$$\begin{aligned} \int _\Omega \nabla u(t,x)\cdot \nabla \varphi (x)\,\text {d}x =\int _\Omega \eta (t,x)\varphi (x)\,\text {d}x\,, \end{aligned}$$

from which it follows that

$$\begin{aligned} -\int _\Omega \Delta u(t,x) \varphi (x)\,\text {d}x +\langle \partial _\mathbf{n}u(t,\cdot ), \varphi _{|\partial \Omega } \rangle _{H^{-1/2}(\partial \Omega ), H^{1/2}(\partial \Omega )} =\int _\Omega \eta (t,x)\varphi (x)\,. \end{aligned}$$

As $-\Delta u = \eta$ in $L^2(0,T; H)$, we infer that

$$\begin{aligned} \langle \partial _\mathbf{n}u(t,\cdot ), \varphi _0 \rangle _{H^{-1/2}(\partial \Omega ), H^{1/2}(\partial \Omega )}=0 \quad \forall \varphi _0\in H^{1/2}(\Omega )\,, \end{aligned}$$

hence $\partial _\mathbf{n}u=0$ almost everywhere in $\Sigma$. Now, since we have that $\Delta u\in L^2(0,T; H)$ and $\partial _\mathbf{n}u =0\in L^2(0,T; H^{1/2}(\partial \Omega ))$, by the elliptic regularity result [41, Thm. 3.2] we infer that $u\in L^2(0,T; W)$. Eventually, letting $\varepsilon \searrow 0$ in the equations (3.14)–(3.15) we obtain

$$\begin{aligned} \partial _t u - \Delta \mu = 0 \quad \text {in } L^2(0,T; H) \end{aligned}$$

and

$$\begin{aligned} \mu =\tau \partial _t u -\Delta u + \xi + \Pi (u)-g \quad \text {in } L^2(0,T; H)\,. \end{aligned}$$

This implies that *u* is a solution to the local Cahn–Hilliard equation according to conditions (3.3)–(3.8), in the viscous case $\tau >0$. This concludes the proof of Theorem 3.3 in the case $\tau >0$.

### The case $\tau =0$

We consider here the case $\tau =0$, so that $\tau _\varepsilon \rightarrow 0$.

We perform the first estimate as in the previous section: we test (3.14) by $\mu _\varepsilon$, (3.15) by $\partial _t u_\varepsilon$, take the difference, and integrate on $Q_t$: we obtain

$$\begin{aligned}&\int _{Q_t}|\nabla \mu _\varepsilon (s,x)|^2\,\text {d}x \,\text {d}s +\tau _\varepsilon \int _{Q_t} |\partial _t u_\varepsilon (s,x)|^2\,\text {d}x \,\text {d}s + E_{\varepsilon }(u_\varepsilon (t,\cdot )) +\\&\quad \int _\Omega (\hat{\gamma }+\hat{\Pi }) (u_\varepsilon (t,x))\,\text {d}x \\&\quad = E_{\varepsilon }(u_{0,\varepsilon })+ \int _\Omega (\hat{\gamma }+\hat{\Pi })(u_{0,\varepsilon }(x))\,\text {d}x +\int _{Q_t}g_\varepsilon (s,x)\partial _t u_\varepsilon (s,x)\,\text {d}x\,\text {d}s\,. \end{aligned}$$

Using now the additional assumption (3.18) in the case $\tau =0$, we can integrate by parts with respect to time in the last term on the right-hand side and use the Young inequality as

$$\begin{aligned}&\int _{Q_t}g_\varepsilon (s,x) \partial _t u_\varepsilon (s,x)\,\text {d}x\,\text {d}s\\&\quad =-\int _{Q_t}\partial _tg_\varepsilon (s,x) u_\varepsilon (s,x)\,\text {d}x\,\text {d}s +\int _{\Omega }g_\varepsilon (t,x) u_\varepsilon (t,x)\,\text {d}x -\int _{\Omega }g_\varepsilon (0,x) u_{0,\varepsilon }(x)\,\text {d}x\\&\quad \leqq \frac{1}{2}\Vert g_\varepsilon \Vert _{H^1(0,T; H)}^2 +\frac{1}{2}\int _{Q_t}|u_\varepsilon (s,x)|^2\,\text {d}x\,\text {d}s +\sigma \int _\Omega |u_\varepsilon (t,x)|^2\,\text {d}x +\frac{1}{4\sigma }\Vert g_\varepsilon (t,\cdot )\Vert _H^2\\&\qquad +\frac{1}{2}\Vert u_{0,\varepsilon }\Vert _H^2 + \frac{1}{2}\Vert g_\varepsilon (0,\cdot )\Vert _H^2 \end{aligned}$$

for every $\sigma >0$. Moreover, note that by the generalized Poincaré inequality contained in [51, Theorem 1.1], there exist constants $C>0$ and $\bar{\varepsilon }\in (0,\varepsilon _0)$, independent of $\varepsilon$ and of *t*, such that

$$\begin{aligned} \int _\Omega |u_\varepsilon (t,x)-(u_\varepsilon (t,\cdot ))_\Omega |^2\,\text {d}x \leqq C E_\varepsilon (u_\varepsilon (t,\cdot )) \quad \forall \,\varepsilon \in (0,\bar{\varepsilon })\,. \end{aligned}$$

Since $(u_\varepsilon )_\Omega =(u_{0,\varepsilon })_\Omega$, rearranging the terms and choosing $\sigma >0$ sufficiently small (independently of $\varepsilon$), we infer that

$$\begin{aligned}&\int _{Q_t}|\nabla \mu _\varepsilon (s,x)|^2\,\text {d}x \,\text {d}s +\tau _\varepsilon \int _{Q_t} |\partial _t u_\varepsilon (s,x)|^2\,\text {d}x \,\text {d}s +E_\varepsilon (u_\varepsilon (t,\cdot )) + \Vert u_\varepsilon (t,\cdot )\Vert _{H}^2 \\&\quad \leqq C\left( E_{\varepsilon }(u_{0,\varepsilon })+ \Vert u_{0,\varepsilon }\Vert _H^2+ \int _\Omega (\hat{\gamma }+\hat{\Pi })(u_{0,\varepsilon }(x))\,\text {d}x +\Vert g_\varepsilon \Vert ^2_{H^1(0,T; H)} \right) \\&\qquad +\int _{Q_t}|u_\varepsilon (s,x)|^2\,\text {d}x\,\text {d}s \end{aligned}$$

for a certain $C>0$ independent of $\varepsilon$. Recalling then the assumptions (3.17)–(3.18), the Gronwall lemma yields

$$\begin{aligned} \Vert \nabla \mu _\varepsilon \Vert _{L^2(0,T; H)} + \Vert u_\varepsilon \Vert _{L^\infty (0,T; V_\varepsilon )} +\tau _\varepsilon ^{1/2}\Vert \partial _t u_\varepsilon \Vert _{L^2(0,T; H)} \leqq C\,, \end{aligned}$$

hence also, by comparison in (3.14),

$$\begin{aligned} \Vert \partial _t u_\varepsilon \Vert _{L^2(0,T; V^*)}\leqq C\,. \end{aligned}$$

At this point, we proceed exactly as in the previous Sect. 5.1, and infer that

$$\begin{aligned} \Vert \xi _\varepsilon \Vert _{L^2(0,T; L^1(\Omega ))}\leqq C\,, \end{aligned}$$

which implies, by comparison in (3.15), that

$$\begin{aligned} \Vert (\mu _\varepsilon )_\Omega \Vert _{L^2(0,T)}\leqq C\,. \end{aligned}$$

We deduce then that

$$\begin{aligned} \Vert \mu _\varepsilon \Vert _{L^2(0,T; V)}\leqq C\,, \end{aligned}$$

and again, by comparison with (3.15) and by the monotonicity of $\gamma$, that

$$\begin{aligned} \Vert B_\varepsilon (u_\varepsilon )\Vert _{L^2(0,T; H)} + \Vert \xi _\varepsilon \Vert _{L^2(0,T; H)}\leqq C\,. \end{aligned}$$

The Aubin-Lions theorems ensure then that, up to not relabeled subsequence, as $\varepsilon \searrow 0$,

5.10 $$\begin{aligned} u_{\varepsilon }\rightarrow u \qquad&\text {in } C^0([0,T]; V^*)\,,\end{aligned}$$

5.11 $$\begin{aligned} u_\varepsilon {\mathop {\rightharpoonup }\limits ^{*}}u \qquad&\text {in } H^1(0,T;V^*)\cap L^\infty (0,T; H)\,, \end{aligned}$$

5.12 $$\begin{aligned} \tau _\varepsilon u_\varepsilon \rightarrow 0 \qquad&\text {in } H^1(0,T;H)\,,\end{aligned}$$

5.13 $$\begin{aligned} B_\varepsilon (u_\varepsilon )\rightharpoonup \eta \qquad&\text {in } L^2(0,T;H)\,,\end{aligned}$$

5.14 $$\begin{aligned} \mu _\varepsilon \rightharpoonup \mu \qquad&\text {in } L^2(0,T;V)\,,\end{aligned}$$

5.15 $$\begin{aligned} \xi _\varepsilon \rightharpoonup \xi \qquad&\text {in } L^2(0,T;H) \end{aligned}$$

for some

$$\begin{aligned} u \in H^1(0,T; V^*)\cap L^\infty (0,T; H)\,, \qquad \mu \in L^2(0,T; V)\,, \qquad \xi ,\eta \in L^2(0,T; H)\,. \end{aligned}$$

Arguing as in the previous Sect. 5.1 thanks to the Lemma 3, the convergence (5.10) and the boundedness of $(E_\varepsilon (u_\varepsilon ))_\varepsilon$ in $L^\infty (0,T)$ imply the strong convergence

$$\begin{aligned} u_\varepsilon \rightarrow u \qquad \text {in } C^0([0,T]; H)\,. \end{aligned}$$

Hence, by the Lipschitz continuity of $\Pi$ we have

$$\begin{aligned} \Pi (u_\varepsilon ) \rightarrow \Pi (u) \qquad \text {in } C^0([0,T]; H)\,, \end{aligned}$$

while the strong-weak closure of $\gamma$ yields $\xi _\varepsilon \in \gamma (u_\varepsilon )$ almost everywhere in *Q*. Moreover, still arguing as in the previous section we obtain that $u\in L^\infty (0,T; V)$, $\eta =-\Delta u$, and $u\in L^2(0,T; W)$ by elliptic regularity.

Passing to the weak limit in (3.14)–(3.15) we obtain then

$$\begin{aligned} \partial _t u - \Delta \mu = 0 \quad \text {in } L^2(0,T; V^*) \end{aligned}$$

and

$$\begin{aligned} \mu = -\Delta u + \xi + \Pi (u)-g \quad \text {in } L^2(0,T; H)\,. \end{aligned}$$

This concludes the proof of Theorem 3.3 also in the case $\tau =0$.
